# An Interplay of S-Nitrosylation and Metal Ion Binding for Astrocytic S100B Protein

**DOI:** 10.1371/journal.pone.0154822

**Published:** 2016-05-09

**Authors:** Małgorzata Bajor, Monika Zaręba-Kozioł, Liliya Zhukova, Krzysztof Goryca, Jarosław Poznański, Aleksandra Wysłouch-Cieszyńska

**Affiliations:** 1 Department of Biophysics, Institute of Biochemistry and Biophysics, Polish Academy of Sciences, Warsaw, Poland; 2 Department of Immunology, Centre for Biostructure Research, Medical University of Warsaw, Warsaw, Poland; 3 Department of Molecular and Cellular Neurobiology, Nencki Institute, Polish Academy of Sciences, Warsaw, Poland; University of Oldenburg, GERMANY

## Abstract

Mammalian S100B protein plays multiple important roles in cellular brain processes. The protein is a clinically used marker for several pathologies including brain injury, neurodegeneration and cancer. High levels of S100B released by astrocytes in Down syndrome patients are responsible for reduced neurogenesis of neural progenitor cells and induction of cell death in neurons. Despite increasing understanding of S100B biology, there are still many questions concerning the detailed molecular mechanisms that determine specific activities of S100B. Elevated overexpression of S100B protein is often synchronized with increased nitric oxide-related activity. In this work we show S100B is a target of exogenous S-nitrosylation in rat brain protein lysate and identify endogenous S-nitrosylation of S100B in a cellular model of astrocytes. Biochemical studies are presented indicating S-nitrosylation tunes the conformation of S100B and modulates its Ca^2+^ and Zn^2+^ binding properties. Our *in vitro* results suggest that the possibility of endogenous S-nitrosylation should be taken into account in the further studies of *in vivo* S100B protein activity, especially under conditions of increased NO-related activity.

## Introduction

Mammalian S100B protein is an important player in a variety of cellular processes. Its biological activity is exhibited through intracellular and extracellular interactions with many different protein targets and has been a subject of extensive studies for over the last 30 years [1]. Intracellularly, a direct role of protein—S100B interactions has been proven for example in the dynamics of cytoskeleton assembly, activity of transcription factors, calcium homeostasis, and cell proliferation and differentiation [1–3]. Extracellular roles for S100B include its interaction with cell surface proteins leading to either neurotrophic or neurotoxic effects depending on the concentration of the protein [4]. S100B is expressed in a variety of cells, the most in brain tissue by astrocytes, oligodendrocytes and Schwann cells but also in melanocytes, myofibers, enteric glial cells, adipocytes, chondrocytes and other (for a comprehensive review see ref. [5–7]). Elevated S100B levels have been detected in brains of patients with Alzheimer disease, Creutzfeld-Jacobs disease, schizophrenia, brain tumors and epilepsy [8–10]. Recently, strong expression and release of S100B has been shown for astroglia obtained from human induced pluripotent stem cells derived from Down Syndrome (DS) patients and related with reduced neurogenesis and increased neuronal cell death [11]. Elevated S100B level is a clinically used diagnostic biomarker for several brain pathologies and melanoma [12–14]. Its contribution to cancer progression, mediated through interaction with the cellular tumor antigen p53, is the basis for increasingly successful, structure based, rational drug design for melanoma treatment [12]. Although the knowledge on the biology of S100B is rapidly increasing, the mechanistic details of its regulation have not been fully elucidated. Metal ion binding and the role of cysteine residues are the two factors mainly discussed in literature that influence the biological activity of S100B. Acetylation of the protein amino terminus is the only detected post translational modification of S100B with unknown function [15].

Ca^2+^ ion is one of the best characterized regulators of S100B activity. There are two pairs of Ca^2+^-binding EF-hand motifs in a homodimer of S100B. One pair of EF-hands consists of a 12 amino acid long C-terminal motif and a second N-terminal site build of 14 amino acids, which is observed only in S100 proteins. Binding of Ca^2+^ induces a conformational rearrangement of the protein [2, 16]. The hydrophobic, helical C-terminal part exposed upon Ca^2+^ binding is one of the sites of interaction with a large number of S100B targets. Ambiguously, Ca^2+^ association constants measured for S100B at physiological ionic strength are in the range of 10^3^ M^-1^ which is too small to explain any biologically relevant Ca^2+^- dependent interactions at the Ca^2+^ concentration *in vivo* [17, 18]. Some factors that increase Ca^2+^ binding to S100B have been already identified *in vitro* such as complex formation with target peptides or covalent derivatization of the cysteine thiols with mercaptoethanol or fluorescence markers [19].

Interactions of S100B with some important targets, e.g. tau protein, may be modulated by Zn^2+^ instead of Ca^2+^ ion binding [20, 21]. An S100B dimer coordinates at least two Zn^2+^ ions with a much higher affinity than it binds Ca^2+^ [22, 23]. Despite an initial hypothesis based on impaired Zn^2+^ binding to the Cys84Ala mutant of S100B, the cysteine thiol group is not involved in Zn^2+^ binding to the protein [21]. Instead, the Zn^2+^-binding sites observed in a crystal structure of a Ca^2+^ and Zn^2+^ loaded S100B were formed at the interface of the protein homodimer by a combination of histidine and carboxylate side chains originating from both S100B subunits [21]. Thus, the strongly decreased Zn^2+^ affinity of Cys84Ala mutant could not be easily explained.

Other roles for cysteine residues of S100B protein have also been suggested. For example, activity of S100B as a neurite extension factor (NEF) was lost when either of the two S100B cysteines was altered by site-directed mutagenesis [24, 25]. Although micromolar concentrations of S100B Cys68ValCys84Ser mutant were able to stimulate glial activation, the 83 stop mutant that did not contain Cys84, was significantly less effective [25]. The important role of cysteines in S100B lead to a hypothesis, which has not yet been properly proven, of the *in vivo* formation of a covalent disulfide S100B dimer.

Development of redox proteomics technologies has shown that cysteines in proteins have very different reactivities. They not only form disulfide bridges but also are targets of modifications by various redox reactive species which are produced in cells during normal function, but may also serve as sensitive, reversible switches in response to different stimuli [26, 27].

Interestingly, in many pathophysiological conditions the high overexpression of S100B protein is synchronized with significantly increased nitric oxide-related activity [28–30]. A relevant physiological consequence of *in vivo* nitric oxide synthases expression and NO production is S-nitrosylation (SNO) of protein cysteine thiols [31, 32]. Elevated nitric oxide synthase activity induces elevated levels of protein S-nitrosylation (protein SNO) [33]. Protein SNO is a reversible posttranslational modification (PTM) which has been shown to regulate signal transduction in diverse tissues including brain [34–36]. Thousands of proteins have been recently identified as SNO targets using proteomic methods [37–40]. Aberrant protein SNO has been documented in human pathologies, e.g. neurodegeneration or cancer [34, 36]. Formation of protein SNO relies on nitric oxide production and other factors including the enzymatic activity of nitrosylating and denitrosylating proteins, redox status of the cell and some still unclear elements defining the susceptibility of a specific protein cysteine to S-nitrosylation [41, 42]. Thus far, there are no reliable methods to theoretically predict sites of posttranslational SNO in proteins, such as those developed for other types of PTM's and only experimental methods are used to predict the susceptibility of a protein cysteine towards this modification.

In our previous work we have revealed that Cys84 in recombinant S100B and Cys85 in another S100 protein family member, recombinant S100A1, are S-nitrosylated in a Ca^2+^- dependent manner by S-nitrosoglutathione (GSNO)—a low-molecular weight endogenous nitrosothiol [43]. Furthermore, we have reported that the Cys85 of S100A1 protein is an endogenous target of S-nitrosylation in PC12 cells [44]. Comparison of structural NMR data for unmodified S100A1SH and S100A1SNO found that the modified Cys85 thiol side chain is involved in a thiol/aromatic molecular switch which changes the conformation and Ca^2+^ ion affinity of S100A1. Based on these results and the sequence similarity of S100 proteins we have proposed that S-nitrosylation may be a regulatory mechanism for a subgroup of S100 proteins with a conserved cysteine residue in their functionally important C-terminal helix.

Recombinant S100BSNO produced by us previously has been shown by van Dieck et al. to modify S100B interaction with peptide fragments of tumor suppressor p53. Nitrosylation increased S100B binding to the p53 C-terminus, but did not affect the second site of interaction—the N-terminal p53 transactivation domain [45].

The goal of this work was to testify whether S100B is susceptible to S-nitrosylation in *in vitro* experimental models of higher biological complexity including cells. This would be an important prediction of the potential biological relevance of S100B S-nitrosylation *in vivo*. The successful detection of S100BSNO inside cells has prompted us to characterize the possible consequences of S-nitrosylation on the properties of S100B protein including metal-binding, which are best known to modulate its interactions with targets. We show that S-nitrosylation of Cys84 increases S100B proteins affinity toward Ca^2+^ and Zn^2+^ ions. Additionally, data are presented based on a non-classical structure elucidation method–the measurement of proton-deuterium exchange rates by mass spectrometry (HDex-MS) that imply that SNO formation fine-tunes the conformation of S100B protein in a metal-free state. Our results suggest that the possibility of endogenous S-nitrosylation should be taken into account in the further studies of *in vivo* S100B protein activity, especially under conditions of increased NO-related activity.

## Materials and Methods

### Animals

The study included wild-type adult male Wistar rats (3 months old) obtained from the Animal House of Polish Academy of Sciences Medical Research Center. All experimental procedures were performed and carried out in accordance with Polish guidelines for care and use of laboratory animals. All animals were housed in the same facility that maintained a 12:12 h light-dark cycle temperatures between 20–26°C, and humidity between 30–70%. In each independent experiment (n = 3), one adult male Wistar rat was sacrificed by decapitation under isoflurane anesthesia and the brain was rapidly removed and kept in HEN buffer (see below) on ice. The experimental protocols were approved by the Local Ethical Committee on Animal Experiments of the Nencki Institute (Permit Number 454/2013) and all efforts were made to minimize animal suffering and to decrease the number of animal used.

### Cell culture experiments

Rat C6 glioma cells (ATCC) were a kind gift of Prof. Jacek Kuznicki (IIMCB, Warsaw, Poland). Cells were cultured at 37°C in a humidified atmosphere containing 5% CO_2_, in DMEM medium (Gibco) with 10% fetal bovine serum (Gibco), 100 U/ml penicillin (Gibco), and 100 U/ml streptomycin (Gibco). Prior analysis intact cells were thoroughly washed with PBS and cultured for additional 24 hours in DMEM medium with only 1% FBS.

### Analysis of exogenous S-nitrosylation in rat brain tissue

Freshly isolated rat brain was homogenized in 10 volumes of HEN buffer (25 mM HEPES pH 7.7, 1 mM EDTA, and 10 μM neocuproine) and centrifuged at 20,000 x *g* for 15 min at 4°C. The supernatant typically contained 5 mg/ml of protein as determined by Bradford assay. 100 μM S-nitrosoglutathione (GSNO) or reduced glutathione (GSH) was added to 250 μl of the total rat brain protein supernatant to make the final concentration of 1 μM of the glutathione derivative in each sample. The reaction mixtures were incubated at 25°C for 20 min. Residual GSNO or GSH were removed by acetone precipitation of proteins. Biotin Switch Technique, as described previously by Jaffrey et al., was used to selectively substitute all S-nitrosylated cysteines present in proteins by a biotin derivative [46]. Total protein fractions after BST were analyzed using reducing 15% Tricine-SDS-PAGE. Selectively biotinylated proteins were captured using streptavidin-HRP conjugated antibodies and visualized using Amersham ECL Western Blotting Detection Reagent (GE Healthcare) with subsequent exposure to X-ray film. Furthermore, S-biotinylated proteins were enriched from the appropriate mixtures using neutravidin-based affinity purification and resolved by reducing 15% Tricine-SDS-PAGE. Gel bands observed in the low molecular weight region (from 6 kDa to 15 kDa) were excised and analyzed. Longer incubation time (16 hours) was used for in-gel tryptic cleavage of proteins taking into account the resistance of S100B to enzymatic digestion [43]. The standard mass spectrometry based protein identification protocol was optimized for the detection of the plausible S100B protein. Measurements were carried using a Nano Aquity Liquid Chromatography system (Waters, Milford, MA) coupled to LTQ-FTICR mass spectrometer (Thermo Scientific). Mascot search engine (version 2.3, MatrixScience, Boston, MA) was used to survey data against UniProtKB/Swiss-Prot database version 2011_07 (529056 sequences). Mascot search parameters were set as follows: taxonomy—Rattus norvegicus, fixed modification—cysteine carbamidomethylation, variable modification—methionine oxidation, parent ion mass tolerance– 30 ppm, fragment ion mass tolerance—0.1 Da, number of missed cleavages—1, enzyme specificity—semi-trypsin.

### Enrichment of endogenously S-nitrosylated proteins using Biotin Switch Technique (BST)

Confluent rat C6 glioma cells resuspended in 250 mM HEPES buffer, pH 7.7 with 1 mM EDTA and 0.1 mM neocuproine (HEN buffer) and homogenized. Total protein concentration of the lysate was adjusted to 1 mg/ml. Obtained cell lysates were treated using BST as previously described [37, 44]. Supernatants from each BST step were collected, proteins were separated using 15% SDS–PAGE and transferred onto PVDF membrane (0.22 μm). After blocking with non-fat dried milk, the PVDF membrane was incubated with goat anti-S100B polyclonal antibody (1:1000 dilution, Santa Cruz Biotechnology) for 1 hour and afterwards for 1 hour with peroxidase-conjugated rabbit anti-goat IgG (1:10000 dilution, Sigma-Aldrich). The peroxidase activity was visualized using the Amersham ECL Western Blotting Detection Reagent (GE Healthcare) with subsequent exposure to X-ray film.

### Expression, purification and chemical S-nitrosylation of recombinant S100B

S100BSH and S100BSNO proteins were obtained according to previously published methods [43, 47]. All details specific for this study together with determination of S100B protein concentration, using amino acid analysis and HPLC methods are presented in the **S1 Text**.

### Zinc binding affinity analysis using a chromogenic chelator—4-(2-pyridylazo)resorcinol (PAR)

Binding of zinc ions to either S100BSH or S100BSNO proteins was determined spectrophotometrically using 4-(2-pyridylazo)resorcinol (PAR) assay [48]. The absorbance for ZnH_x_PAR_2_ complex was determined at 500 nm using Cary UV-Visible spectrophotometer (Varian) with quartz cuvettes of 1 cm path length. 10 μM S100BSH or S100BSNO proteins were titrated with 0–60 μM ZnSO_4_ in presence of 64 μM PAR. Control titrations were performed for 64 μM PAR in different experimental buffer. The absorption spectra were collected from 200 to 600 nm at 25°C. Experiments were performed in Chelex^®^100-treated buffers as follows: 10 mM TES, pH 7.2, 15 mM NaCl; 10 mM TES, pH 7.2, 10 mM CaCl_2_, 15 mM NaCl; and 10 mM TES, 150 mM NaCl, pH 7.2. Relationship between the concentration of the zinc ion associated with the complex and the absorbance at 500 nm served as a qualitative estimation of zinc affinity to S100B protein variants.

### Isothermal Titration Calorimetry (ITC) experiments

For detailed preparation of buffers and protein solutions for ITC experiments see the **S1 Text**. ITC measurements were carried out at 25°C using Microcal OMEGA ultrasensitive titration calorimeter (MicroCal Inc.). Titration parameters (the number, volume and length of time of injections) were set by the software program controlling data. Solutions in the cell were stirred by a syringe at 400 rpm. The sample cell (1.3611 cm^3^) contained S100B protein solution, while the reference cell contained only buffer. Upon equilibration, a calcium chloride solution prepared in the same buffer as used in the sample cell, or zinc sulfate solution in pure water, was injected in 40 x 4 μL aliquots using the default injection rate. 180 s intervals between each injection allowed the sample to return to baseline. When required, additional experiments were carried out on nanoITC calorimeter (TA Instruments) using 62 injections of 4 μL to the 0.95 cm^3^ sample cell. Integrated heat effects of each injection were corrected by subtraction of the corresponding integrated heat effects of CaCl_2_ (or ZnSO_4_) injection to the pure buffer and heat effects of buffer injection to the protein solution.

### Numerical methods used for ITC data analysis

Instead of iterative numerical models used in original Microcal/TA software provided by the instrument producers, analytical models were used to fit experimental ITC data as described by us previously [49, 50] and details in the **S1 Text**. Simplest binding models that reproduce appropriate experimental data are shown in **Table 1**. Symbol 2s describes two sequential binding sites, 2i - two independent binding sites (which is equivalent to a single binding site of the occupancy n = 2), 2i+2i, a combination of two sets of independent binding sites, that substantially differ in their thermodynamic properties, each of occupancy equal 2, and 2s+2i a combination of two sequential binding sites with the third site (of occupancy equal 2) independent from the previous two. In general, Zn-binding to “strong” (either 2s or 2i type) and “weak” (if detectable solely 2i) binding sites are independent. The thermodynamic parameters, together with their standard errors, were estimated as the average values obtained from at least three independent experiments (with the only exception of Ca^2+^-binding at high-salt conditions). The putative stoichiometry for the Ca^2+^ binding by S100B-SH at high-salt was estimated using the method originally proposed by Job [51].

**Table 1:** ITC-derived thermodynamic parameters for Ca^2+^ binding to S100BSH and S100BSNO protein monomers.

Protein	site	n	Model	Kas*	∆H (kcal/mol)	T∆S (kcal/mol)	∆G (kcal/mol)	∆S (cal/mol/K)
**S100BSH (low salt)**	Ca1	1	2s	0.3–8.9 10^4^	2.5±0.8	8.3±1.0	-5.8±1.0	27.8±3.3
	Ca1+Ca2	2		0.2–3.5 10^9^	7.5±1.0	19.7±0.7	-12.2±0.9	66.1±2.4
	Ca2	1		0.24–1.1 10^5^	5.0±0.2	11.4±0.5	-6.5±0.4	38.3±1.6
**S100BSNO (low salt)**	Ca1	1	2s	0.02–1.1 10^6^	-0.3±1.6	6.8±2.5	-7.1±1.2	22.8±8.6
	Ca1+Ca2	2		0.16–4.5 10^10^	2.5±0.7	16.1±0.6	-13.6±1.0	54.1±2.1
	Ca2	1		0.31–1.1 10^5^	2.8±1.6	9.3±1.9	-6.5±0.4	31.3±6.5
**S100BSH (high salt)**	Ca1,2	2^x^	2i	0.9–2.6 10^4^	1.2±0.3	7.0±0.4	-5.8±0.3	23±1.0
**S100BSNO (high salt)**	Ca1	1	2s	0.4–1.1 10^4^	0.1±0.3	5.4±0.4	-5.3±0.3	18±1.0
	Ca1+Ca2	2		0.77–2.1 10^7^	3.2±1.0	13.0±1.0	-9.8±0.3	44±4.0
	Ca2	1		0.9–3.8 10^3^	3.1±1.0	7.6±1.1	-4.5±0.4	25±4.0

* Range of values estimated from at least three independent experiments, [M^-1^] for K_as_, [M^-2^] for K1_as_ K2_as_

^X^ Stoichiometry assumed according to Job Plot (see **S7 Fig**)

### Hydrogen-Deuterium Exchange Mass Spectrometry

Comparative hydrogen deuterium exchange mass spectrometry experiments were performed for 100 μM solutions of recombinant S100BSH and S100BSNO proteins as described previously [52]. The peptides displaying decreased H/D exchange in S100BSNO are mapped on the solution NMR structure of rat apo-S100B (PDB #1B4C) [53, 54].

## Results

### Identification of S100B protein as a potential S-nitrosylation target in rat brain

Biotin switch technique is the mainstay experimental method to detect protein SNOs in complex biological systems [46, 55]. BST is based on using ascorbate for selective reduction of the protein SNO moiety to a reduced thiol group (in the presence of other thiol modifications), derivatization of the released thiol by a biotin derivative. Biotinylated protein fraction is enrichment by affinity chromatography using neutravidin beads and further analyzed. In the pioneer publication describing development of BST it has been shown that treating the whole brain protein lysate with 1 μM or less nitric oxide donor leads to selective S-nitrosylation of some brain proteins, that have been afterwards detected as S-nitrosylated *in vivo* [56]. Only proteins of molecular mass higher than 20 kDa were analyzed. In this work we used analogous experimental conditions to induce protein S-nitrosylation using GSNO but employed higher density (>15%) SDS-PAGE gels to analyze the GSNO-treated brain lysate fraction enriched by BST (**S1 Fig**). Using such procedure, routine for the detection of small S100 proteins, we have observed proteins of low molecular mass that were not detected previously. Gel bands observed in the low molecular weight region were excised, washed and digested with trypsin. Resulting peptides were recovered by extraction and analyzed by mass spectrometry. S100B protein has been clearly identified in the analyzed fractions. MS/MS fragmentation spectra for the Cys containing S100B peptide are shown in **S1 Fig**.

### S100B protein is endogenously S-nitrosylated in C6 glioma cells

**Fig 1:**
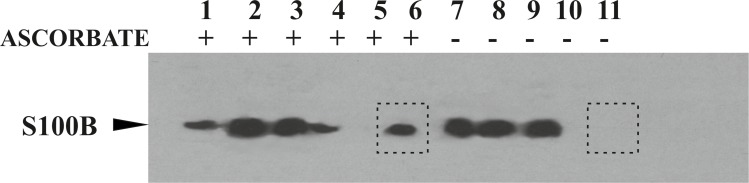
S100B protein is S-nitrosylated in non-stimulated C6 glioma cells. The presence of S100B in protein fractions collected from each step of BST was determined by Western blotting. Chemically *S*-nitrosylated, recombinant human S100BSNO protein eluted from neutravidin resin after BST enrichment, a positive control of BST *(lane 1);* total C6 glioma lysate before BST *(lane 2)*, fully derivatized protein fraction before affinity enrichment on neutravidin resin *(lane 3)*; protein fraction unbound to neutravidin *(lane 4*); resin wash fraction (*lane 5*); proteins enriched on neutravidin resins *(lane 6);* total C6 glioma protein lysate before BST *(lane 7*); total protein fraction before affinity enrichment on neutravidin resin (*lane 8*); protein fraction unbound to neutravidin *(lane 9*); wash fraction (*lane 10);* proteins nonspecifically enriched on neutravidin resins, negative control of BST *(lane 11)*. *Lanes 1–6* depict BST with ascorbate reduction and *lanes 7–11* without ascorbate reduction step. A clear difference in the presence of S100B band in the protein fraction enriched on neutravidin beads after BST procedure with (*lane 6*) and without (*lane 11*) ascorbic acid is marked by rectangles.

We employed C6 glioma cells, a commonly used experimental model of astrocytes, to prove the hypothesis that S100B protein may be endogenously S-nitrosylated inside cells. SNO proteins from whole cell lysates of confluent, unstimulated C6 glioma cells were enriched using optimized BST as described by us previously [37, 44]. Control experiments, in which ascorbate reduction of SNO bonds was eliminated, were performed to identify proteins that nonspecifically bind to neutravidin resin. Recombinant S100BSNO protein was used as a positive control of the BST efficiency in our hands. Protein fractions obtained at the end step of BST were analyzed by Western blotting by S100B recognizing antibody. As marked in **Fig 1**, S100B has been detected in the positive control experiment **(Fig 1, lane 1)**, the fraction enriched by a full BST protocol that included ascorbate reduction **(Fig 1, lane 6)**, and is not observed in the negative control samples **(Fig 1, lane 11)**. Such result unambiguously indicates that S-nitrosylation is an intracellular modification of S100B protein.

### Synthesis and initial characterization of unmodified and S-nitrosylated recombinant human S100B protein

In order to gain insight into the consequences of S-nitrosylation on intrinsic properties of S100B, both unmodified and S-nitrosylated recombinant human S100B forms have been obtained and purified to homogeneity, as described by us previously [43] and in the **S2 Fig**. Purity and correct masses of intact proteins were confirmed by analytical HPLC and mass spectrometry, respectively (**S3 and S4 Figs**). Similarity of CD spectra profiles for both variants suggested they both fold into similar structures (**S5 Fig**). Size exclusion chromatography profiles show that under all experimental conditions used in this work both S100B protein variants were stable homodimers (**S6 Fig**).

### Calcium affinity of S100B protein is increased by SNO both under high and low ionic strength conditions

**Fig 2:**
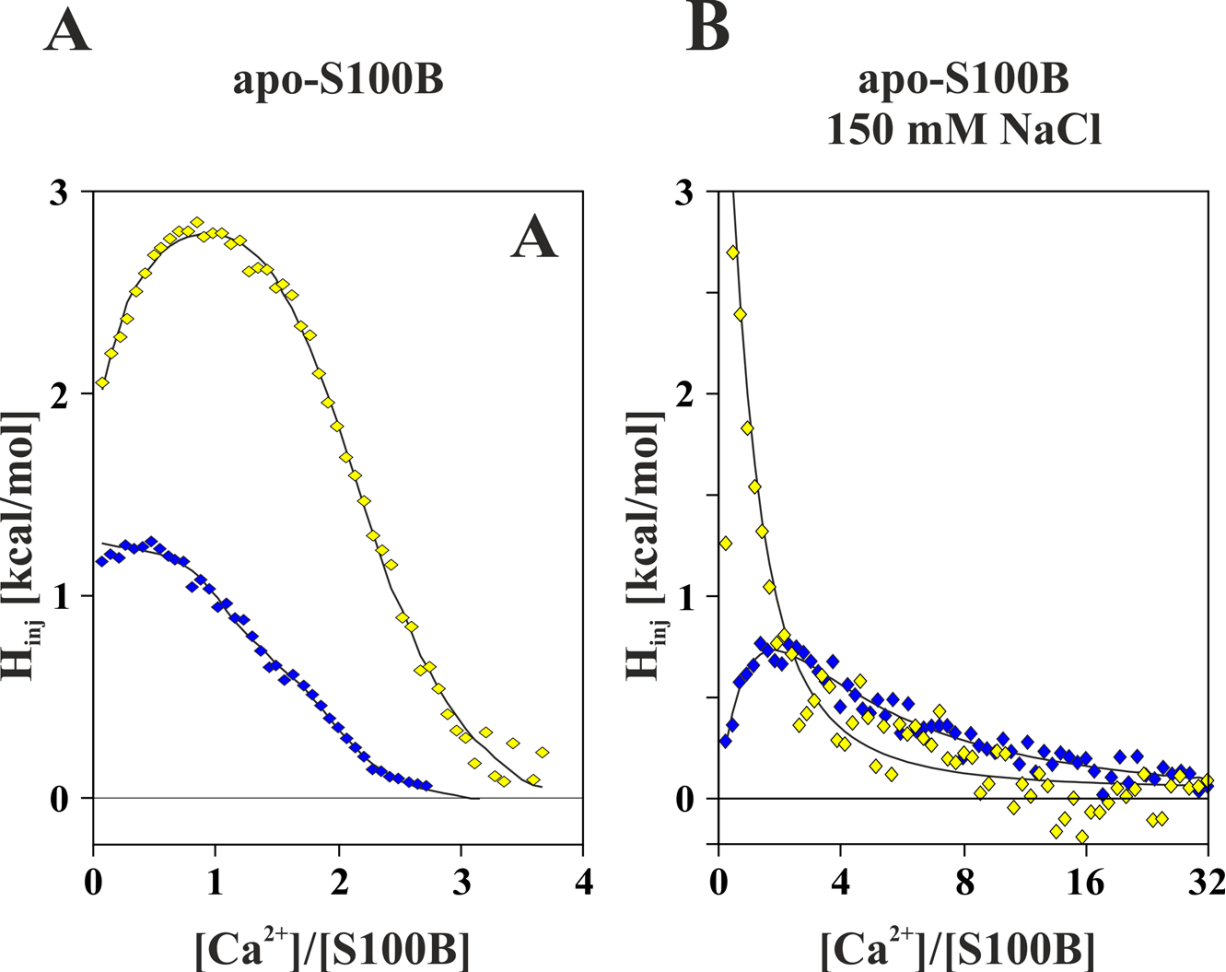
Representative integrated heat plots obtained for ITC titrations of Ca^2+^ ions to S100BSH (yellow diamonds) and S100BSNO (blue diamonds) protein solutions in TES buffer, pH 7.2 at 25°C containing either 15 mM (A) or 150 mM (B) NaCl. The lines following data points represent best-fitted models described in **Table 1**.

Ca^2+^ ion binding is a prerequisite for interaction of S100B protein with many biological targets. In order to detect if S-nitrosylation attenuates Ca^2+^ affinity to S100B protein we used homogenous solutions of recombinant S100BSH and its S100BSNO counterpart and performed comparative isothermal titration calorimetry (ITC) analysis of Ca^2+^ binding to these proteins. ITC experiments were performed under two experimental conditions in TES buffer, pH 7.2, with either low (15 mM) or high (150 mM) NaCl concentration at 25°C. The integrated heat flow of individual injections (binding isothermograms) for representative CaCl_2_ titration of S100BSH and S100BSNO at low (**Fig 2A**) and high (**Fig 2B**) ionic strength are presented in **Fig 2**as a function of the Ca^2+^ to protein monomer molar ratio.

Presented data reveal that under all measured conditions Ca^2+^-binding is an endothermic, entropy driven process for both variants of S100B. The shape of all obtained Ca^2+^ binding curves are similar except for unmodified S100B protein under higher ionic strength conditions. Although, the exponential decay observed in the latter curve could suggest that only a single Ca^2+^ is bound by the protein, a Job Plot analysis of data (**S7 Fig**) supports a 2:1 stoichiometry for Ca^2+^/S100BSH binding. Consequently, approximately four Ca^2+^ ions are coordinated by an S100B dimer (two per monomer) in all cases, regardless the salt concentration and the presence of S-nitrosylation. Under low salt conditions, the unfavorable total heat effect accompanying Ca^2+^ binding is nearly 3 times lower for S100BSNO than for S100BSH, strongly facilitating Ca^2+^ binding. **Table 1**shows calculated thermodynamic parameters for Ca^2+^ binding to the investigated S100B forms. The binding isotherms were fit using a model of either sequential or independent binding of two cations per monomer (2s or 2i in **Table 1**).

Average association constants for Ca^2+^-binding obtained by us for unmodified S100BSH in low salt are in close agreement with data previously published by others (3.6 10^4^ M^-1^ and 55.6 10^4^ M^-1^, for S100BSH EF-hand 1 and EF-hand 2 motif, respectively) [19, 57]. Under our experimental conditions, the SNO modification in S100BSNO strengthens the overall binding of Ca^2+^ cations to the protein by an order of magnitude from a measured range of K_Ca1+Ca2_ from 0.2–3.5 10^9^ M^-2^ to 0.16–4.5 10^10^ M^-2^. As indicated by a different shape of the initial part of the binding isotherm this change is mainly due to a significant increase of the firstly bound Ca^2+^ affinity, while binding of the second Ca^2+^ ion is virtually unaffected. The significant increase of Ca^2+^ affinity of the first EF-hand of S100BSNO results both from favorable enthalpy and entropy changes (TΔΔS_SNOCa1_ = 1.5 kcal/mol and ΔΔH_SNOCa1_ = -2.8 kcal/mol), whereas the binding of the succeeding ion is almost unaltered due to a compensation of the SNO-related enthalpy and entropy changes (TΔΔS_SNOCa2_ = -2.1 kcal/mol, ΔΔH_SNOCa2_ = -2.2 kcal/mol). Consistent with previously published data [58] presence of a monovalent Na^+^ cation in the protein solutions lead to a three orders of magnitude decrease of Ca^2+^ binding affinity for S100B in comparison to low salt conditions (**Table 1**). Interestingly, ITC data clearly indicate that higher concentration of Na^+^ makes binding of the two Ca^2+^ ions to an S100BSH monomer completely independent. This suggests no interaction between the two EF-hand binding sites. Such effect is not observed when Cys84 of S100B is S-nitrosylated.

### S-nitrosylation is important in multifactorial regulation of Zn^2+^ binding to S100B

**Fig 3:**
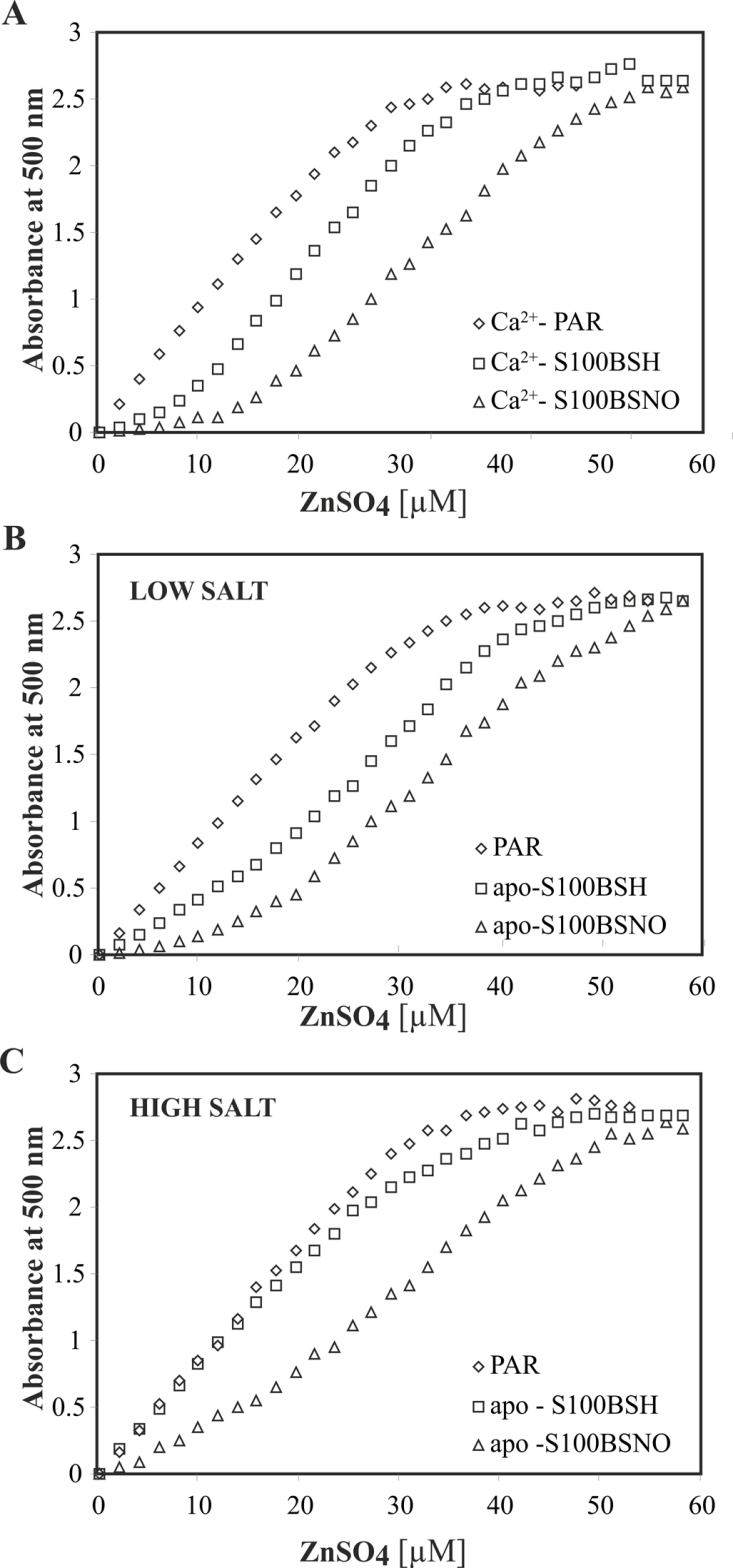
Titration curves of PAR alone (diamonds) and in presence of either S100BSH (squares) or S100BSNO (triangles) proteins with ZnSO_4_ measured in absorbance at 500 nm at 25°C. Experiments were performed at (A) 10 mM TES buffer, pH 7.2, 10 mM CaCl_2_, 15 mM NaCl; (B) 10 mM TES buffer, pH 7.2, 15 mM NaCl and (C) 10 mM TES buffer, pH 7.2, 150 mM NaCl.

Despite Ca^2+^-based modulation, the activity of S100B protein may be regulated *in vivo* by Zn^2+^ binding. To analyze the effect of S-nitrosylation on Zn^2+^ affinities to S100B, we initially performed a competition assay with a chromogenic Zn^2+^ ion chelator 4-(2-pyridylazo)resorcinol (PAR). **Fig 3**shows the absorbance of the formed ZnH_x_PAR_2_ complex detected at 500 nm for 64 μM PAR chelator itself, a mixture of 64 μM PAR and 10 μM S100BSH protein, and a mixture of 64 μM PAR and 10 μM S100BSNO, as a function of total Zn^2+^ in three different solutions (TES buffer, pH 7.2 containing either CaCl_2_ (A) or 15 mM NaCl (B), or 150 mM NaCl (C)).

As recently presented by Kocyła et al. [59] the use of PAR for indirect studies of zinc binding to metalloproteins is not straightforward and strictly relies on proper knowledge of the formed ZnH_x_PAR_2_ complex stability, its molar absorption coefficient and the stoichiometry of the Zn^2+^-metalloprotein interaction, all of which may depend on the content of experimental buffers. Thus, in this work we have compared only spectrophotometric data obtained in buffers of the same composition. Decreased absorbance of the ZnH_x_PAR_2_ chromophore in the presence of S100B variants indicates Zn^2+^ binding to the proteins. Under all of the conditions Zn^2+^ affinity was higher for S100BSNO than for S100BSH.

**Fig 4:**
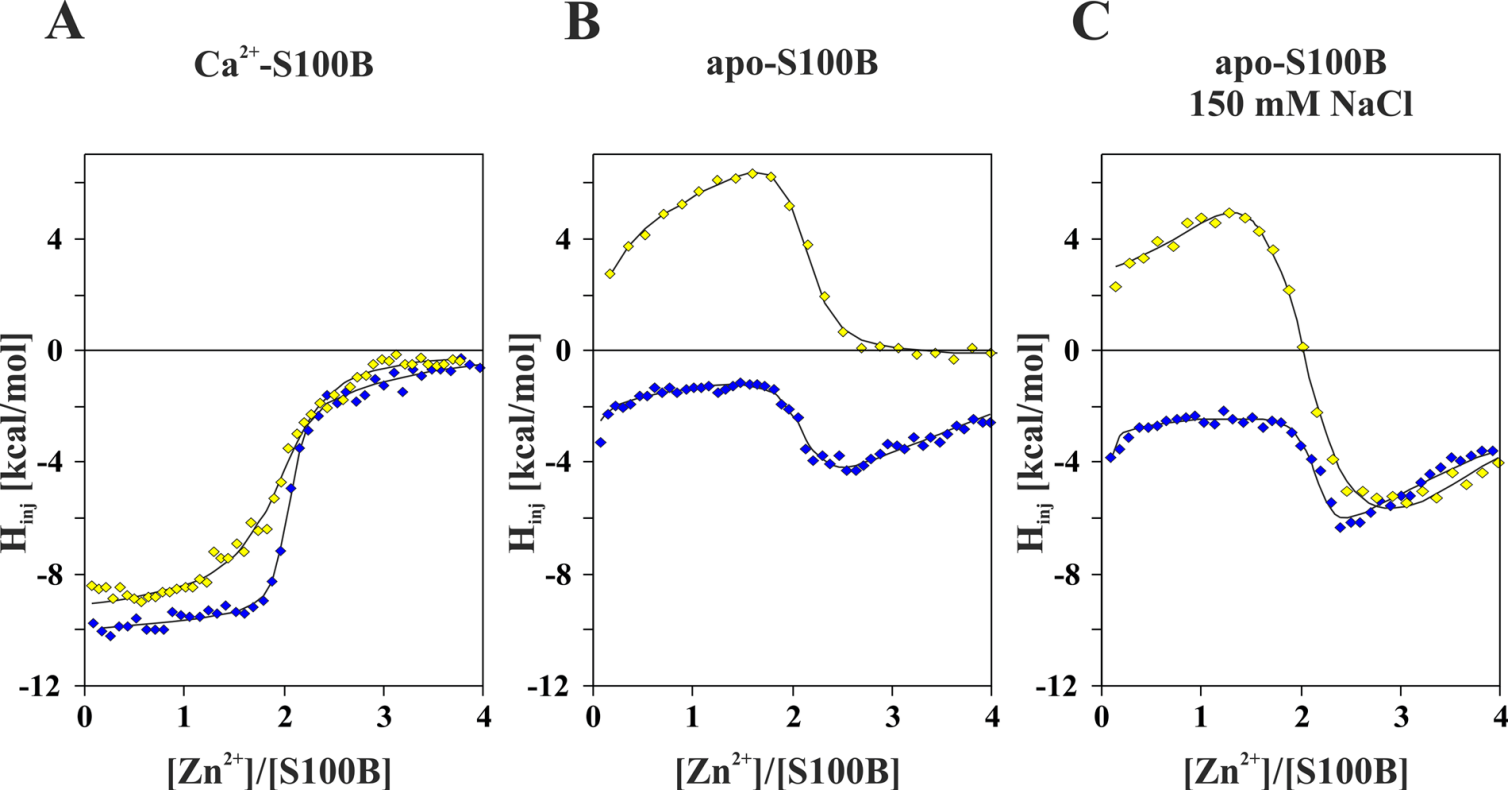
Representative integrated heat plots obtained for ITC titrations of Zn^2+^ ions to S100BSH (yellow diamonds) and S100BSNO (blue diamonds) protein solutions. (A) Integrated heat plots for *holo* Ca^2+^-S100BSH and *holo* Ca^2+^-S100BSNO protein with ZnSO_4_ in 10 mM TES buffer, pH 7.2, 15 mM NaCl. (B) Integrated heat plots for *apo* S100BSH and *apo* S100BSNO protein with ZnSO_4_ in 10 mM TES buffer, pH 7.2, 15 mM NaCl. (C) Integrated heat plots for *apo* S100BSH and *apo* S100BSNO protein with ZnSO_4_ in 10 mM TES buffer, pH 7.2, 150 mM NaCl.

Direct ITC titration experiments, similar to described above for Ca^2+^ binding studies, were used to obtain precise numerical values of the Zn^2+^ to S100B affinity constants. The influence of SNO and Ca^2+^-loading on Zn^2+^-binding to S100B was investigated. To achieve the goal, comparative titrations were performed for both S100BSH and S100BSNO under several experimental settings including the *apo* proteins under low and high salt conditions (15 mM or 150 mM NaCl, respectively), and for the fully Ca^2+^ loaded (*holo*) variants. **Fig 4A–4C**show representative experimental Zn^2+^-binding isotherms obtained in our study. Usually three experiments were performed for a different combination of S100B variant and buffer composition. Zn^2+^ salt concentration in the titrant was varied if necessary to obtain high precision data points in different parts of the binding curves. All of the binding isothermograms obtained in this study are collected in **S8 Fig**.

As can be clearly observed in **Fig 4**, the isothermograms differed qualitatively from each other depending on the presence of SNO, the loading of S100B with Ca^2+^, as well as the NaCl concentration in the buffer. The most striking observed difference is the change from strongly exothermic Zn^2+^ binding by the Ca^2+^ -loaded proteins to endothermic reactions for the Ca^2+^-free S100B forms. Irrespective of ionic strength conditions, noncovalent dimers of both Ca^2+^-loaded (*holo*) S100BSH and S100BSNO proteins strongly bind two Zn^2+^ ions independently (model 2i in **Table 2**). However, two additional, weaker binding independent sites can be identified for S100BSNO (model 2i+2i). The Zn^2+^ binding parameters for unmodified *holo* S100BSH (K_SHCa,Zn_ = 1.3–5.3 10^6^ M^-1^) are close to data published previously (1.06 ± 0.18 10^7^ M^-1^) [22]. Interestingly, *holo* S100BSNO binds Zn^2+^ more than 30 folds stronger than S100BSH with K_SNOCa,Zn_ in the range of 3.5–9.2 10^7^ M^-1^. This is clearly evidenced in **Fig 4A** by a much sharper transition observed at [Zn^2+^] to [S100B] ratio of 2. For both Ca^2+^-loaded proteins, Zn^2+^ binding is mostly exothermic with a very low entropic contribution (ΔS_SNOCa,Zn_, see **Table 2**). The additional, approximately three orders of magnitude weaker binding sites detected solely for *holo* S100BSNO are entropy driven. Tightening of Zn^2+^-binding for *holo* S100B due to SNO of Cys84 thiol is opposite to its decrease observed previously after thiol side chain removal by a Cys84Ala mutation of S100B [22].

**Table 2:** ITC-derived thermodynamic parameters for Zn^2+^ binding to S100BSH and S100BSNO protein dimer.

Protein	site	n	Model	Kas*	∆H(kcal/mol)	T∆S(kcal/mol)	∆G(kcal/mol)	∆S(cal/mol/K)
***holo* S100BSH**	Zn1,2	2	2i	1.3–5.3 10^6^	-9.0±1.0	-0.2±0.5	-8.8±0.4	-1.0±2.0
***holo* S100BSNO**	Zn1,2	2	2i+2i	3.5–9.2 10^7^	-9.2±1.2	1.5±1.4	-10.6±0.3	4.9±4.6
	Zn3,4	2		0.3–3.1 10^5^	-3.3±1.4	3.5±2.0	-6.80±0.7	11.7±6.8
***apo* S100BSH(low salt)**	Zn1	1	2s	2.3–3.4 10^6^	0.7±1.0	9.5±1.0	-8.8±0.1	32±3.4
	Zn1+Zn2	2		3.5–5.6 10^12^	10.2±0.9	27.6±0.7	-17.4±0.1	93±2.5
	Zn2	1		1.4–1.8 10^6^	9.6±1.6	18.1±1.6	-8.5±0.1	61±5.3
***apo* S100BSNO(low salt)**	Zn1	1	2s+2i	*nd*	*nd*	*nd*	*nd*	*nd*
	Zn1+Zn2	2		0.6–7.6 10^13^	-1.4±0.7	16.9±0.1	-18.3±0.8	56.7±0.3
	Zn2	1		*nd*	*nd*	*nd*	*nd*	*nd*
	Zn3,4	2		0.4–1.7 10^5^	-6.1±1.6	0.7±2.1	-6.7±0.4	2.3±6.9
***apo* S100BSH(high salt)**	Zn1	1	2s+2i	1.6–2.5 10^7^	3.5±1.0	13.5±1.1	-10.0±0.1	45±4.0
	Zn1+Zn2	2		2.0–4.9 10^13^	11.9±0.5	30.4±0.3	-18.5±0.3	102±1.0
	Zn2	1		1.0–2.8 10^6^	8.3±1.3	16.9±1.2	-8.6±0.3	57±4.0
	Zn3,4	2		0.8–2.8 10^5^	-8.0±2.6	-0.9±3.0	-7.1±0.4	-3.0±10.0
***apo* S100BSNO(high salt)**	Zn1	1	2s+2i	*nd*	*nd*	*nd*	*nd*	*nd*
	Zn1+Zn2	2		0.9–1.4 10^13^	2.1±7.0	20.0±7.0	-17.9±0.1	67±23.0
	Zn2	1		*nd*	*nd*	*nd*	*nd*	*nd*
	Zn3,4	2		0.3–1.6 10^5^	-6.8±3.2	-0.2±3.7	-6.6±0.5	-0.7±13.0

* Range of values estimated from at least three independent experiments, [M^-1^] for K_as_, [M^-2^] for K1_as_ K2_as_

*nd*–not determined

Zn^2+^ binding to metal-free (*apo*) S100B has not yet been studied by ITC. Zinc salt—induced S100B oligomerization and precipitation has been suggested previously [22]. Based on our experimental experience we have assumed that protein precipitation may be due to incremental amounts of Zn(OH)_2_ precipitates in buffered solutions of ZnSO_4_. The differences between heat effects of injecting aqueous and buffered ZnSO_4_ solutions to the proteins was insignificant. Indeed, if pure aqueous solutions of ZnSO_4_ were used for ITC titrations we were able to successfully obtain Zn^2+^ binding isothermograms for Ca^2+^-free (*apo*) S100BSH and S100BSNO in the presence of low (15 mM) and high (150 mM) NaCl concentrations in the buffer (presented in **Fig 4B and 4C**, respectively). Substantial differences in the shape of measured isotherms have been related both to changes in the ionic strength of the buffer and to the presence or absence of the SNO modification. In contrast to data presented above for *holo* proteins, the titration curves for *apo* S100B forms are not monotonic, but show one, for S100B in low salt (15 mM NaCl), or even two inflection points, clearly identifying a contribution from two or three types of binding sites, respectively. The experimental data were best reproduced with a model that assumed two, unequivalent, sequential Zn^2+^ binding sites per an S100B dimer, possibly accompanied by two weaker ones, which are independent both from the first two, and from each other (model 2s+2i, **Table 2**). This qualitatively differs from the observations made for *holo* forms of S100BSH and S100BSNO, in which the independent binding of the two first Zn^2+^ cations was observed (models either 2i or 2i+2i in **Table 2**). Thus, in the absence of Ca^2+^, the Zn^2+^ sites in S100B interact with each other, leading to the sequential, highly cooperative binding of the ions. As shown in **Table 2**, S-nitrosylation of Ca^2+^-loaded S100B leads to a gain in the free energy of binding of the first two Zn^2+^ cations. A similar, but slightly lower effect, is also observed for *apo* protein in low salt conditions, although not as pronounced as for the Ca^2+^- loaded S100B.

In conclusion, the ITC-derived data reveal that the unmodified S100B exists as an equilibrium of Zn^2+^-free, single Zn^2+^ (dominating form) and doubly Zn^2+^-loaded protein populations, while only the *apo* and doubly Zn^2+^-loaded populations exist for S100BSNO, due to highly cooperative binding of the two strongly bound Zn^2+^ ions. This cooperativity is even stronger at higher ionic strength of the buffer (150 mM NaCl). Thus, in a buffer expected to more closely resemble the physiological conditions, posttranslational SNO allows for a more efficient Zn^2+^-dependent regulation of S100B already at moderate Zn^2+^ concentration.

### Mass spectrometry monitored hydrogen/deuterium exchange used to analyze SNO-induced effects in S100B protein

**Fig 5:**
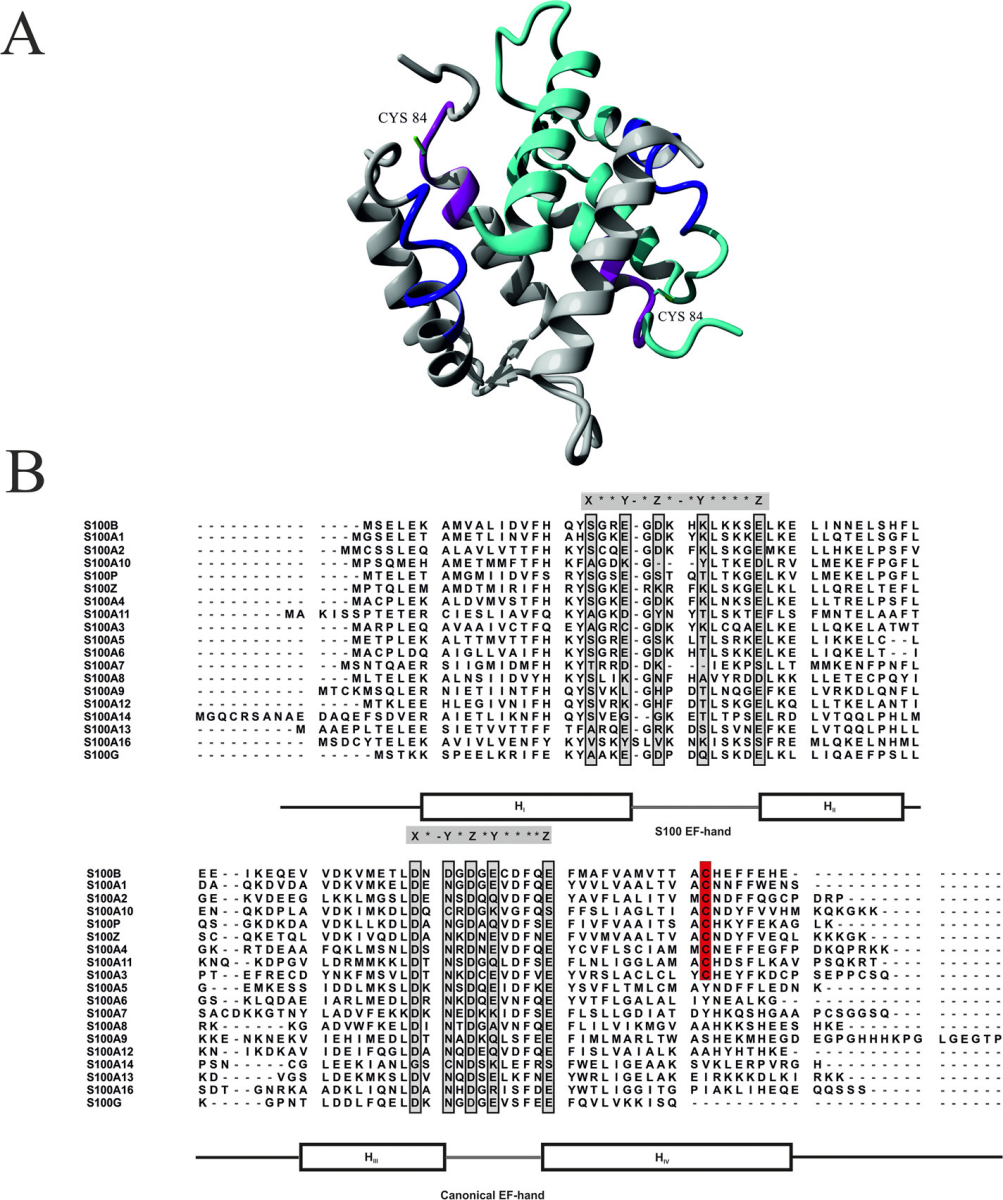
(A) Peptides displaying decreased H/D exchange in S100BSNO mapped on a NMR solution structure of rat *apo*-S100B protein (PDB #1B4C). One of S100B protein monomers is marked in grey, cysteine 84 side chains are marked in green. Peptide fragments Val80-Glu86 and Leu35-Phe43 are marked in magenta and navy, respectively. (B) Sequence alignment of human S100 proteins. Conserved carboxyterminal Cys residues are highlighted in red. Residues responsible for Ca^2+^ coordination in the EF-hand loops are marked light grey.

Rates of proton/deuterium (H/D) exchange of amide protons in S100BSH and S100BSNO proteins were measured by mass spectrometry under identical experimental conditions, in which the integrity and stability of the protein homodimers remained similar (data not shown). Average changes of masses related to different rates of deuterium incorporation were measured for peptic fragments of both S100B variants. They were minimal for most fragments except for two peptides: Val80-Glu86 and Leu35-Phe43 for which the HD exchange was significantly slower in S100BSNO than in S100BSH (presented in **Table 3)**. Higher protection of amide hydrogens in these regions of S100BSNO suggests an SNO-induced formation of a population of more stiffened, hydrogen bonded structures directly in the vicinity of S-nitrosylation (Cys84) and in the linker loop that connects the two EF-hand type calcium binding domains in S100B protein. Though linker region is far in the protein sequence from the modification site, it is proximal in space to Cys84 and may be affected by the presence of the SNO group. **Fig 5A** shows the protein fragments of SNO-induced reduced flexibility marked on the NMR structure of a rat S100B protein.

**Table 3:** H/D exchange results for S100BSH and S100BSNO as assessed by liquid chromatography combined with mass spectrometry (LC-MS).

Residue number	Peptide sequence	m/z (charge)	Mass [Da]	H/D exchange (mass shift) after 10 min		H/D exchange (mass shift) after 30 min	
				S100BSH	S100BSNO	S100BSH	S100BSNO
35–43	LINNELSHF	543.8(2)	1085.6	5.16±0.28	4.52±0.22	5.84±0.17	4.80±0.18
80–86	VTTACHE	760.5(1)	759.5	5.88±0.31	4.50±0.13	7.20±0.13	5.13±0.14

## Discussion

S100 family proteins are multifunctional molecules with regulatory roles in a variety of physiological and pathological processes. This lead to a long discussed question of how the individual family members are regulated to play specific biological roles. The biological activity of S100 proteins is mainly achieved through target binding, often in a metal ion—dependent way in response to increased Ca^2+^- or Zn^2+^- ion levels. Knowledge on other regulatory mechanisms of S100 activities, including posttranslational modifications is still very limited. Most of the known S100 proteins, except S100A12, S100A13 and calbindin have cysteine residues in their primary sequence (**Fig 5B**). Redox modifications of cysteine thiols, in particular posttranslational S-nitrosylation has been shown important in neutrophil S100 proteins, S100A8 and S100A9. S-nitrosylation of S100A8 has been revealed to regulate inflammatory processes [60, 61]. Recently, it has been detected that upon inflammatory stimulus of a mixture of interferon gamma and oxidatively-modified low-density lipoprotein a heterodimer of S100A8 and S100A9 plays a crucial role in an S-nitrosylase complex with the inducible form of nitric oxide synthase (iNOS) [62]. The presence of both S100A8 and S100A9 is responsible for selectivity of the complex, while a cysteine of S100A9 is S-nitrosylated by iNOS and transfers the NO group to cysteines in selected protein targets i.e. GAPDH [62]. Endogenous S-nitrosylation of another S100 protein family member, the cardiac and brain S100A1, has been detected in PC12 cells [44].

In this work we experimentally revealed that a cysteine thiol in S100B protein is one of the targets of S-nitrosoglutathione induced S-nitrosylation in whole rat brain lysate. This is similar to several other proteins identified in a similar experimental setting previously by Jaffrey et al. (58). Most of these proteins were later identified to be important nitrosylation targets *in vivo* [56]. Furthermore, we detected S-nitrosylation as an endogenous PTM of S100B protein in C6 glioma cells—a frequently used experimental model of astrocytes. This is only the second identified to date PTM of S100B. The presence of S100BSNO inside cells motivated us to study the molecular mechanism by which S-nitrosylation may differentiate the activity of S100B protein.

It is not fully understood how post translational S-nitrosylation exerts its effects in proteins. The simplest identified mechanism is direct chemical protection of a cysteine thiol. This way SNO derivatization of active site thiols alters the activity of enzymes such as dimethylarginine dimethylaminohydrolase [63]. However, no Cys-dependent enzymatic activity has been assigned to S100B. Under other circumstances, reversible formation of SNO has been shown to temporarily shield reactive protein thiols from irreversible oxidation under oxidative stress [64] or on the contrary activate the thiol toward formation of further post-translational modifications like sulphenic or sulphonic acid as for human glutathione reductase [65]. Information on the structural consequences of SNO adduct formation in proteins is very limited. There are only 14 X-ray structures of SNO modified proteins deposited in the PDB database for 8 unique proteins due to difficulties in obtaining homogenous, selectively S-nitrosylated proteins in sufficient amounts for structural studies and the lability of SNO bond because of its sensitivity to radiation. In most of the solved structures replacement of a cysteine free thiol by the SNO group does not lead to conformational changes other than accommodation of the NO group close to the site of modification. However, in thioredoxin 1 the SNO of Cys62 results in a significant helix rotation and structure disordering [66], while in protein tyrosine phosphatase PTP1B the SNO of Cys215 leads to a reorganization of hydrogen bond network [64]. This indicated a role for allosteric mechanisms in the SNO-related control of protein function. Comparison of structural NMR data for unmodified S100A1 and S100A1SNO, performed by us previously, found that the Cys85 thiol side chain forms a thiol/aromatic molecular switch which upon S-nitrosylation changes the conformation of S100A1. Another effect of S100A1 S-nitrosylation was an increased calcium affinity of the protein.

Data presented in this work provide detailed information on the consequences of S-nitrosylation and the unique flexibility of human S100B protein in changing its modes of binding and affinities toward Ca^2+^ and Zn^2+^ ions under different conditions. Various experimental techniques have been used by others to reveal metal-binding properties of S100 proteins including S100B [57, 67, 68]. In our work we initially tried to use optical spectroscopic techniques to study the influence of posttranslational SNO on metal ion binding to S100B. Unfortunately, neither UV-vis (data not shown) or CD spectra differentiated significantly the Ca^2+^ bound and free forms of S100BSH and S100BSNO. Other commonly used methods of measuring metal ion to protein binding are competition assays with relatively cheap chromogenic chelators of appropriate ions. To obtain reliable results using such methods an optimal chelator should not interact with the protein, has to have well defined stoichiometries of the formed metal ion complexes, and metal ion affinities close to the proteins studied [59, 69]. As the Ca^2+^ affinities of S100B are known to differ over three orders of magnitude under the low and high ionic strength conditions used in this work we did not attempt to use the chelator studies in this case. However, using the water soluble Zn^2+^ chelator—4-(2-pyridilazo) resorcinol (PAR) we initially revealed that S-nitrosylation significantly increases Zn^2+^ affinity of S100B protein.

Isothermal titration calorimetry has been used previously to study both metal ion binding to S100 proteins and their mutants [22]. ITC is particularly well suited to study the thermodynamic consequences of PTMs of proteins [70, 71]. Its superiority over other methods is that ITC experiments are performed in solution, do not require additional protein labeling, and directly provide thermodynamic data concerning the stoichiometry (n), association constants (Kas) and changes of standard molar enthalpy (ΔH). Thus, we used ITC as a direct method to compare the Ca^2+^ and Zn^2+^ binding to S100BSH and S100BSNO in further detail. ITC-derived data presented herein provide a multitude of different information. Detailed information on the thermodynamic parameters of binding, including in most cases both cumulative binding constants and the binding affinities of individual ions has been obtained. Presented data confirm the large decrease in Ca^2+^ affinity for S100B protein in buffers that contain 150 mM NaCl relative to low salt buffers. Since the binding of the two Ca^2+^ ions to unmodified S100B under high ionic strength conditions is totally independent the possible molecular mechanism of such a decrease is the lack of contact between the Ca^2+^ binding sites in S100B. Interaction of pairs of EF-hand sites has been proven important for efficient ion binding in many EF-hand type proteins [72]. Presented data indicate that S-nitrosylation significantly tightens Ca^2+^ binding to S100B under all conditions studied and thus if present under native conditions could lead to a greater proportion of Ca^2+^-loaded protein capable of interacting with its targets.

In addition to Ca^2+^—binding modulation, SNO of Cys84 significantly increases the Zn^2+^-affinity of S100B (up to two orders of magnitude for the Ca^2+^-loaded form) under various experimental conditions. Calculated binding constants and thermodynamic parameters indicate a significant change in the mode of Zn^2+^ binding for the Ca^2+^-loaded S100B, which is not identical in unmodified and S-nitrosylated S100B forms. While interrelation of Ca^2+^ and Zn^2+^ binding has been previously described for unmodified S100B and other S100 proteins, which are known to bind both of these ions [22, 67, 73], this work adds the new information on modulation of this cross-talk by posttranslational S-nitrosylation of S100B.

Until now, a connection of S-nitrosylation and Zn^2+^-binding has been only described for proteins in which Zn^2+^ is directly coordinated by cysteine thiol ligands, like metallothionein or zinc finger domains. SNO of the coordinating thiols impairs Zn^2+^ binding and leads to release of the ion or strong weakening of its binding [74]. To the best of our knowledge, S100B is the first example of a protein in which regulation of Zn^2+^-binding may be directly connected to a reversible PTM of a non-coordinating cysteine residue. Our data suggest that nitrosylation of proteins may serve as an NO-dependent, reversible molecular mechanism of transferring of the Zn^2+^ ion from strong protein binders, with thiols in the primary Zn^2+^ coordination sphere, which are significantly weakened by thiol SNO, to weaker binders with SNO-induced, increased Zn^2+^ affinity, such as S100B protein, which coordinate Zn^2+^ by a combination of His and Glu side chains and may be present *in vivo* in very high concentrations.

Previous studies described the use of engineered mutants of S100 proteins to find a relationship between protein sequence, structure and ion binding in this class of proteins. For some of the mutant proteins, structural perturbations were observed far from the site of mutation. This lead to a hypothesis that the four-helix EF-hand domains is as a single globally cooperative unit regulated by some key residues that are crucial for the protein's fold and function [75]. HD exchange experiments presented in this work indicate that SNO of Cys84 induces conformational rearrangements in S100B at two sites in the protein sequence, the linker region and the C-terminal helix, both important for S100Bs interaction with biological targets. This suggests that Cys84 is one of the key regulatory residues in the EF-hand type S100B protein. Similar results have been previously obtained for the S100A1 protein dimer by classical structure elucidation studies using NMR spectroscopy.

In conclusion, based on our *in vitro* studies, it is tempting to propose that formation of endogenous SNO of S100B protein could be one of the *in vivo* mechanisms that, through regulation of the proteins affinity to metal ions, and modulation of protein conformation at structural elements that are crucial for target binding, could be responsible for the functional diversity of the protein.

Further *in vivo* research is necessary to prove the role of S-nitrosylation of S100B i.e. under conditions when nitrosative stress accompanies a very high overexpression of S100B protein as observed in brain pathologies like Down Syndrome or Alzheimer’s disease and also in other cell types and other pathologies. For example, NO synthase activity is strongly up-regulated in melanoma cell lines, while the concentration of S100B in melanocytic tumor is roughly 100 times higher than in normal skin [76]. If confirmed, the endogenous formation of S100BSNO in melanoma cells would have be taken under consideration in the on-going drug discovery process for melanoma treatment based on small molecule inhibitors directly targeting S100B [2].

## Supporting Information

S1 Fig Detection of protein S-nitrosylation in a brain lysate. Brain lysates were treated with NO donor GSNO analyzed using BST. Modified proteins were enriched using neutravidin resin and analyzed using tricine-SDS-PAGE. Excised from the gel protein bands followed by trypsin digestion were measured using Nano Aquity Liquid Chromatography system (Waters) coupled to LTQ-FTICR mass spectrometer (Thermo Scientific). (A) Western blot analysis of biotinylated proteins in GSH- (lane 1) and GSNO-treated (lane 2) brain lysate. Proteins after BST were resolved by tricine-SDS-PAGE, transferred to PVDF membranes, and detected using anti-biotin antibody. (B) Tricine-SDS-PAGE gel of SNO proteins after BST, enriched using neutravidin resin in GSH- (lane 1) and GSNO-treated (lane 2) brain lysate. (C) Annotated MS/MS-derived sequence of S100B peptide.
(PDF)

S2 Fig Preparative purification of recombinant S100B protein variants. (A) Representative preparative chromatograms obtained for isolation of S100BSH protein from E. coli bacterial culture by semi-preparative HPLC (C18 column, 45 to 65% mobile phase (0.1% TFA in acetonitrile (v/v) in 60 min; flow rate: 2 mL/min). The protein elution was detected by UV simultaneously at two different wavelengths either 220 and 280 nm. (B) Representative chromatograms obtained for S-nitrosylated S100B protein by semi-preparative HPLC (C18 column, 50 to 58% mobile phase (0.1% TFA in acetonitrile (v/v) in 40 min; flow rate: 2 mL/min). The protein elution was detected by UV simultaneously at two different wavelengths either 220 and 334 nm.
(PDF)

S3 Fig Representative analytical chromatograms of S100BSH and S100BNO proteins after dialysis prior the ITC runs. The reversed-phase gradient for analysis was from 50 to 64% mobile phase (0.1% TFA in acetonitrile (v/v)) in 14 min; flow rate: 1 mL/min. The protein elution was detected by UV simultaneously at two different wavelengths either 220 and 280 nm for S100BSH (A) or 220 nm and 334 nm for S100BSNO (B).
(PDF)

S4 Fig Mass spectrometry analysis of S100B proteins. ESI mass spectrum before (A, B) and after deconvolution (C, D) for S100BSH (left panel) and S100BNO (right panel) proteins using Q-TOF Premier mass spectrometer.
(PDF)

S5 Fig **Far UV CD spectra of the *apo* (A) and *holo* (B) S100BSH (black solid line) and S100BSNO (blue dash line) proteins.** CD spectra were collected using Aviv Circular Dichroism Spectrometer Model 202, with quartz cuvettes of 0.1 cm path length at 25°C. Measurement was performed for each protein at 10 μM concentration in 10 mM TES buffer, pH 7.2, an average of three scans was recorded scanning from 198 nm to 250 nm.
(PDF)

S6 Fig Oligomeric state of S100BSH and S100BSNO in solution determined by size exclusion chromatography. Proteins were analyzed before (black lines) and after (red lines) every ITC run. 200 μl of 100 μM protein solution was loaded onto Superdex 75 10/300 GL size exclusion chromatography column (GE Healthcare) in appropriate buffers (as indicated at chromatograms A-H). Proteins were eluted as one oligomeric species corresponding to dimeric form of S100B protein both for unmodified and SNO variants.
(PDF)

S7 Fig Job plot for the Ca^2+^ binding to the S100BSH protein in high ionic strength buffer. Job plot suggesting the binding stoichiometry for an Ca^2+^-S100BSH complex in 10 mM TES buffer, pH 7.2, 150 mM NaCl.
(PDF)

S8 Fig Ca^2+^ and Zn^2+^ binding to the recombinant S100B protein variants. All ITC data (binding isothermograms) obtained for titration of: Ca^2+^ ions to S100BSH (A1-A7) and S100BSNO (B1-B3) protein solutions in TES buffer, pH 7.2, 15 mM NaCl at 25°C; Ca^2+^ ions to S100BSH (C1) and S100BSNO (D1) protein solutions in TES buffer, pH 7.2, 150 mM NaCl at 25°C; Zn^2+^ ions to S100BSH (E1-E3) and S100BSNO (F1-F3) protein solutions in TES buffer, pH 7.2, 15 mM NaCl at 25°C; Zn^2+^ ions to S100BSH (G1-G3) and S100BSNO (H1-H2) protein solutions in TES buffer, pH 7.2, 150 mM NaCl at 25°C; Zn^2+^ ions to Ca^2+^-S100BSH (I1-I3) and Ca^2+^-S100BSNO (J1-J3) protein solutions in TES buffer, pH 7.2, 15 mM NaCl at 25°C.
(PDF)

S1 Text Materials and Methods. (DOCX)
