# Supplementary figures and images for: An Interplay of S-Nitrosylation and Metal Ion Binding for Astrocytic S100B Protein
Source: PLoS One. 2016 May 9;11(5):e0154822. doi: 10.1371/journal.pone.0154822 (PMC4861259; doi:10.1371/journal.pone.0154822)

**A**

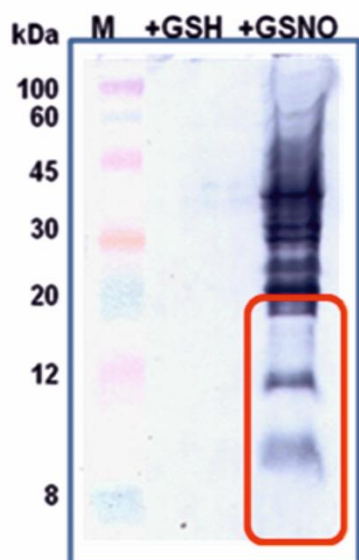

**B**

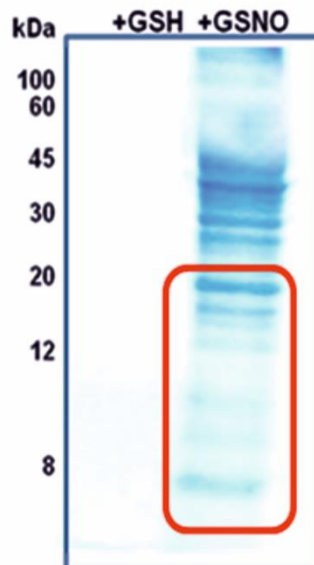

**C**

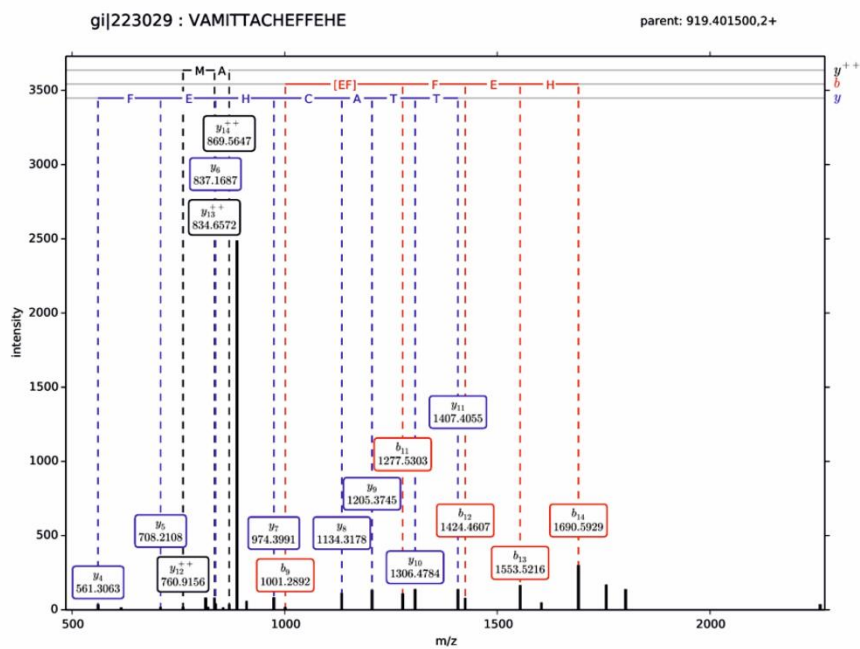

Supplement: S1 Fig — Brain lysates were treated with NO donor GSNO analyzed using BST. Modified proteins were enriched using neutravidin resin and analyzed using tricine-SDS-PAGE. Excised from the gel protein bands followed by trypsin digestion were measured using Nano Aquity Liquid Chromatography system (Waters) coupled to LTQ-FTICR mass spectrometer (Thermo Scientific). (A) Western blot analysis of biotinylated proteins in GSH- (lane 1) and GSNO-treated (lane 2) brain lysate. Proteins after BST were resolved by tricine-SDS-PAGE, transferred to PVDF membranes, and detected using anti-biotin antibody. (B) Tricine-SDS-PAGE gel of SNO proteins after BST, enriched using neutravidin resin in GSH- (lane 1) and GSNO-treated (lane 2) brain lysate. (C) Annotated MS/MS-derived sequence of S100B peptide. (PDF) [file pone.0154822.s001.pdf]

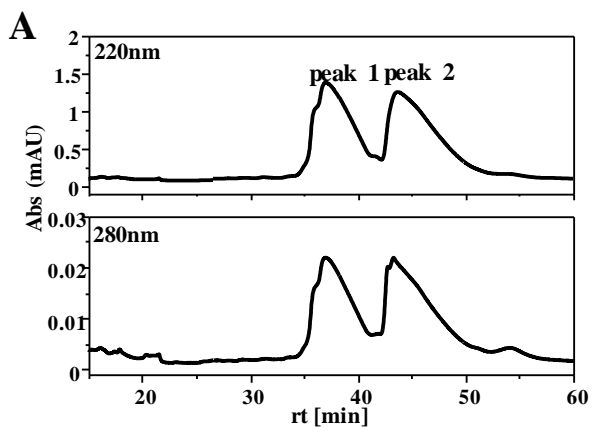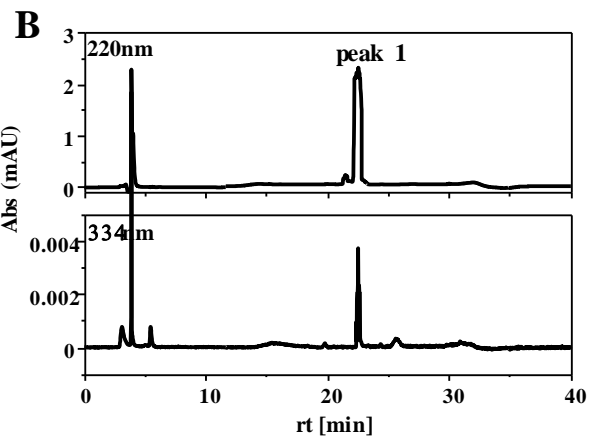

Supplement: S2 Fig — (A) Representative preparative chromatograms obtained for isolation of S100BSH protein from E. coli bacterial culture by semi-preparative HPLC (C18 column, 45 to 65% mobile phase (0.1% TFA in acetonitrile (v/v) in 60 min; flow rate: 2 mL/min). The protein elution was detected by UV simultaneously at two different wavelengths either 220 and 280 nm. (B) Representative chromatograms obtained for S-nitrosylated S100B protein by semi-preparative HPLC (C18 column, 50 to 58% mobile phase (0.1% TFA in acetonitrile (v/v) in 40 min; flow rate: 2 mL/min). The protein elution was detected by UV simultaneously at two different wavelengths either 220 and 334 nm. (PDF) [file pone.0154822.s002.pdf]

A

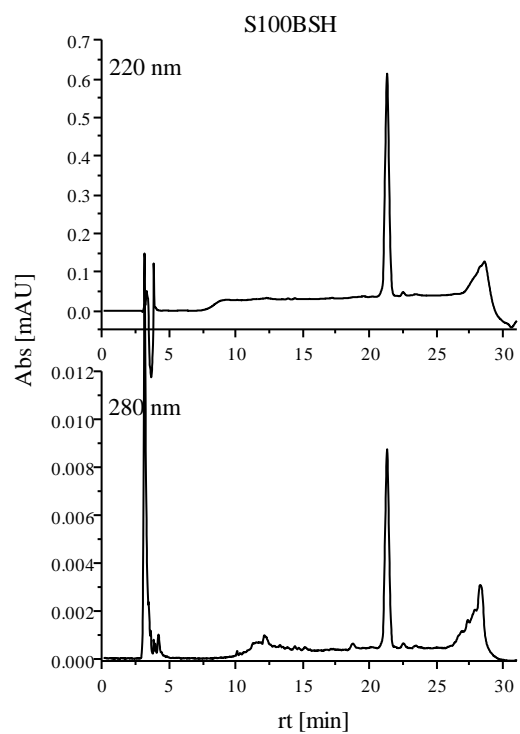

B

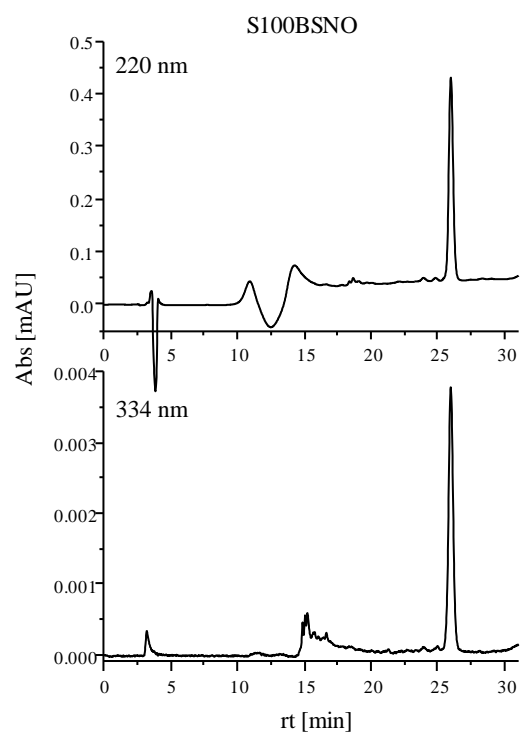

Supplement: S3 Fig — The reversed-phase gradient for analysis was from 50 to 64% mobile phase (0.1% TFA in acetonitrile (v/v)) in 14 min; flow rate: 1 mL/min. The protein elution was detected by UV simultaneously at two different wavelengths either 220 and 280 nm for S100BSH (A) or 220 nm and 334 nm for S100BSNO (B). (PDF) [file pone.0154822.s003.pdf]

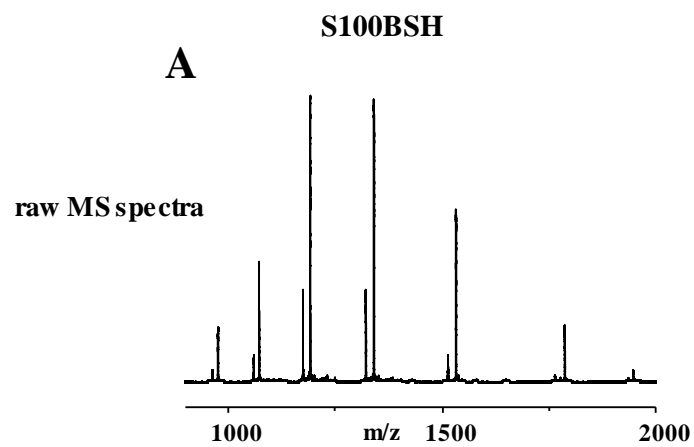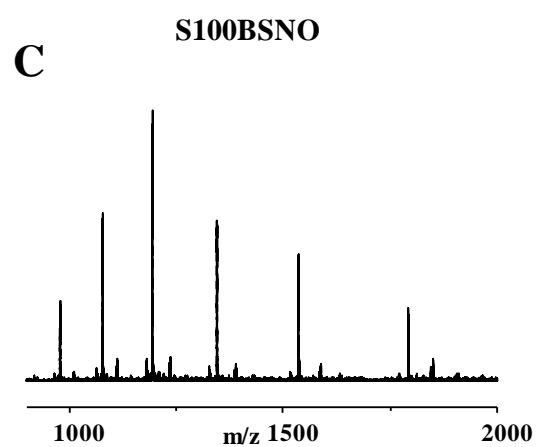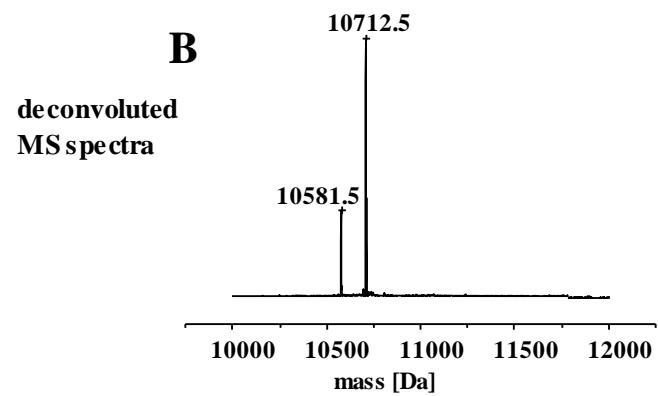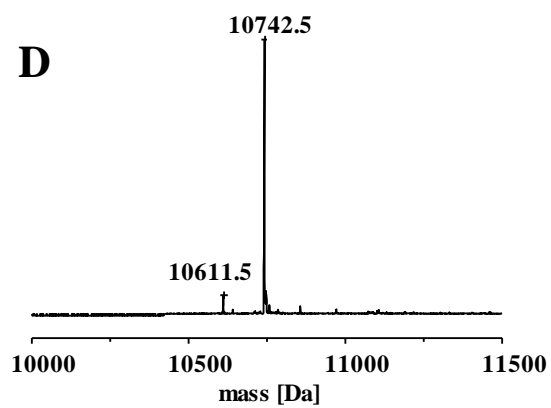

Supplement: S4 Fig — ESI mass spectrum before (A, B) and after deconvolution (C, D) for S100BSH (left panel) and S100BNO (right panel) proteins using Q-TOF Premier mass spectrometer. (PDF) [file pone.0154822.s004.pdf]

A

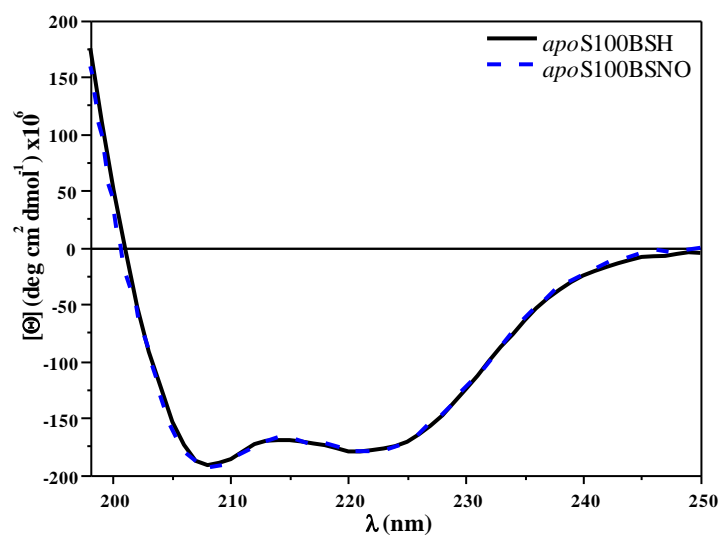

B

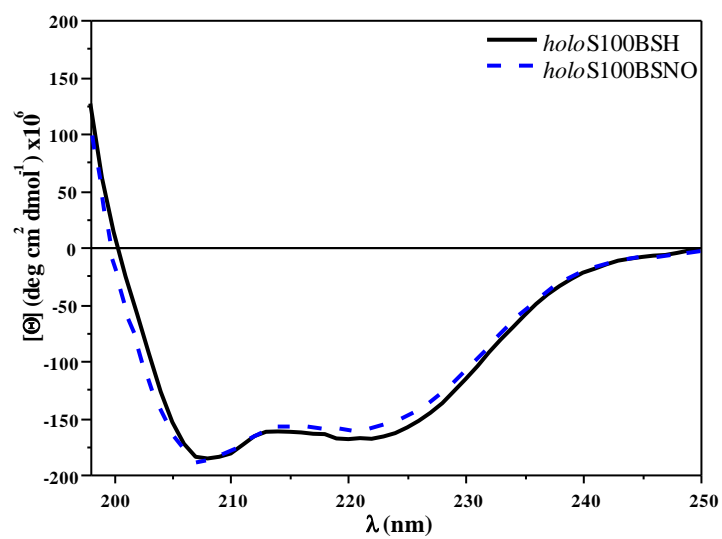

Supplement: S5 Fig — Far UV CD spectra of the apo (A) and holo (B) S100BSH (black solid line) and S100BSNO (blue dash line) proteins. CD spectra were collected using Aviv Circular Dichroism Spectrometer Model 202, with quartz cuvettes of 0.1 cm path length at 25°C. Measurement was performed for each protein at 10 μM concentration in 10 mM TES buffer, pH 7.2, an average of three scans was recorded scanning from 198 nm to 250 nm. (PDF) [file pone.0154822.s005.pdf]

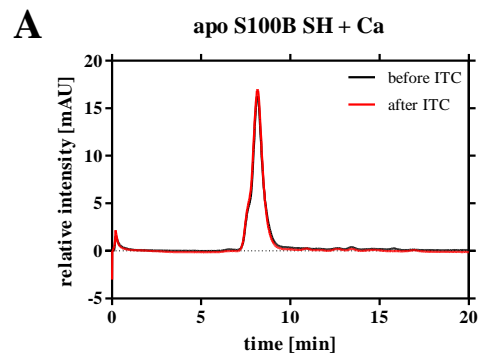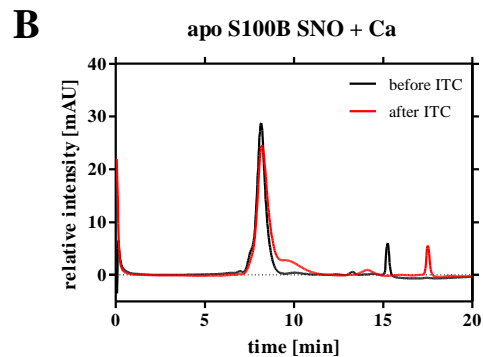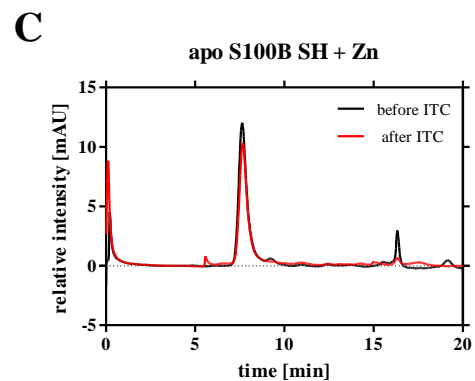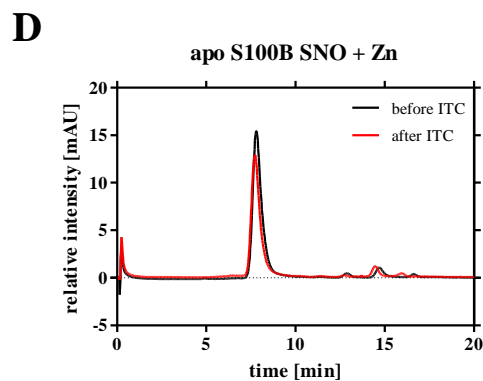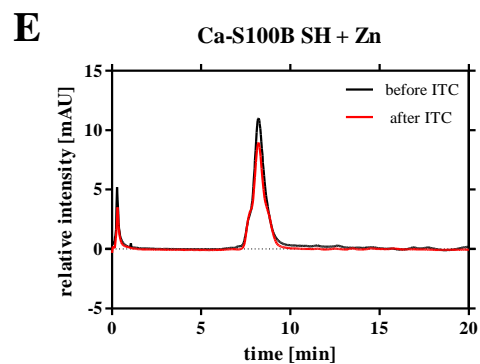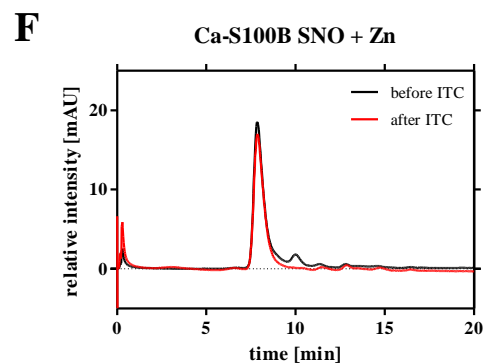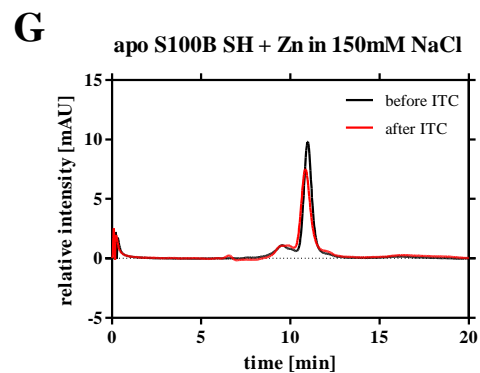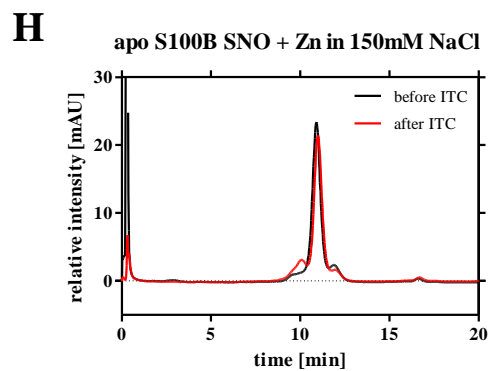

Supplement: S6 Fig — Proteins were analyzed before (black lines) and after (red lines) every ITC run. 200 μl of 100 μM protein solution was loaded onto Superdex 75 10/300 GL size exclusion chromatography column (GE Healthcare) in appropriate buffers (as indicated at chromatograms A-H). Proteins were eluted as one oligomeric species corresponding to dimeric form of S100B protein both for unmodified and SNO variants. (PDF) [file pone.0154822.s006.pdf]

apo-S100BSH  
150 mM NaCl

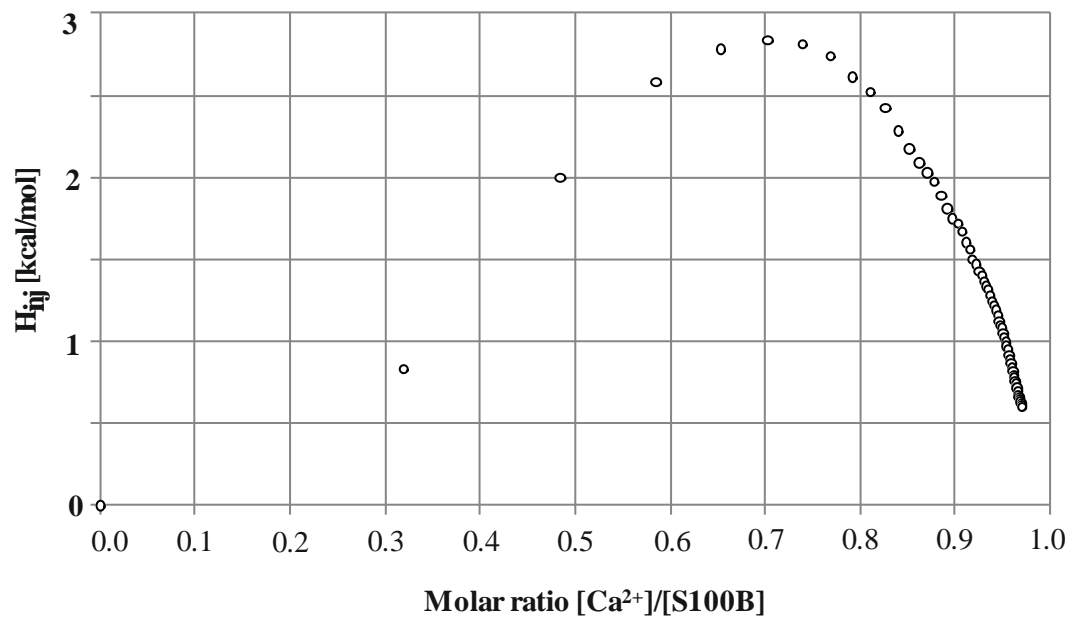

Supplement: S7 Fig — Job plot suggesting the binding stoichiometry for an Ca2+-S100BSH complex in 10 mM TES buffer, pH 7.2, 150 mM NaCl. (PDF) [file pone.0154822.s007.pdf]

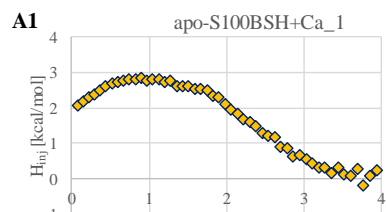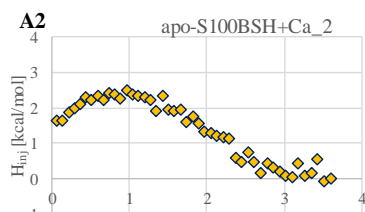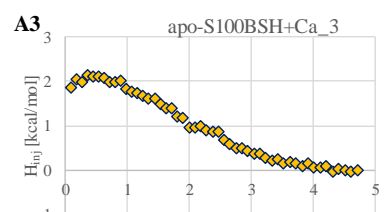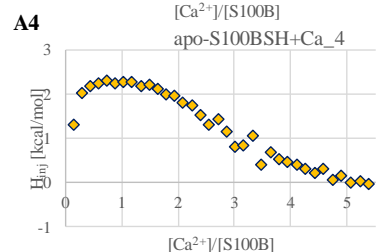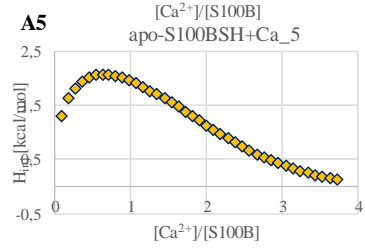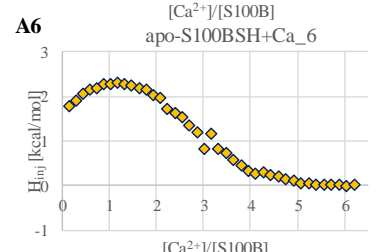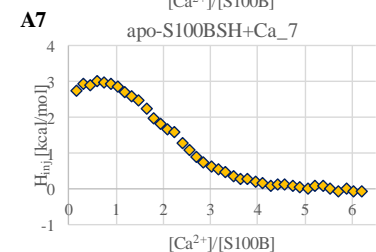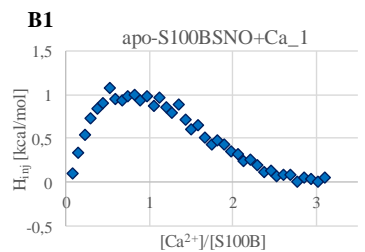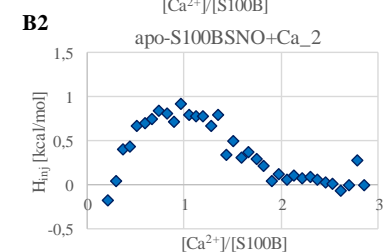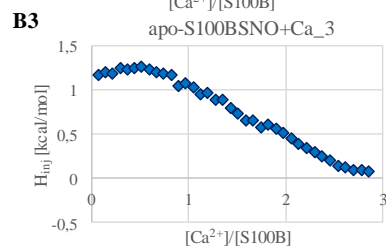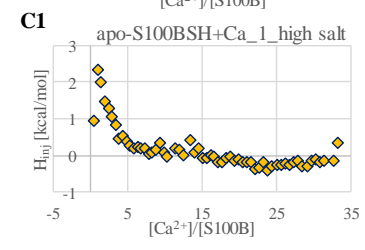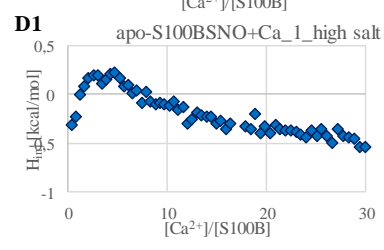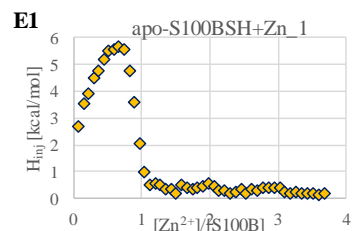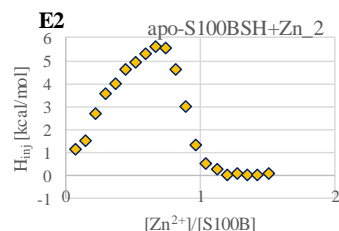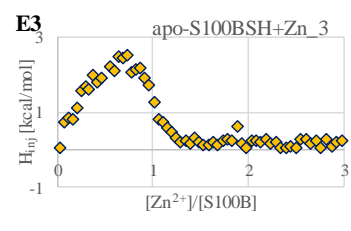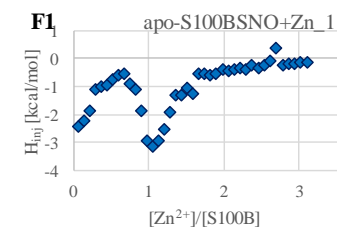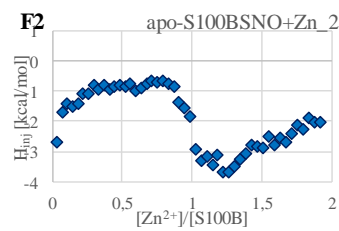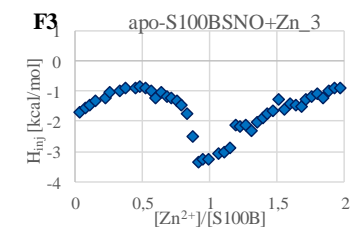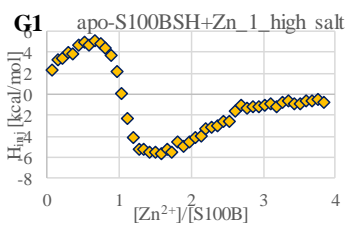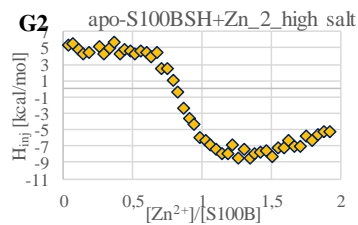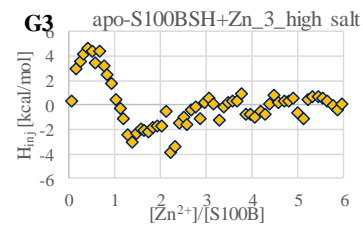

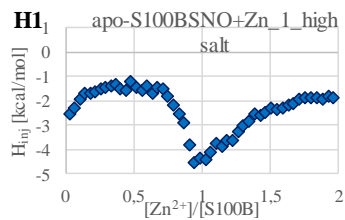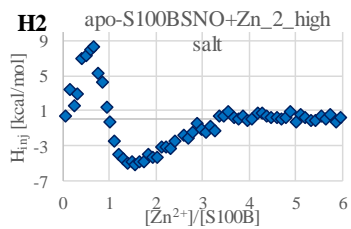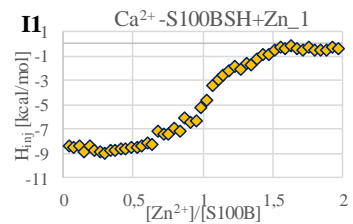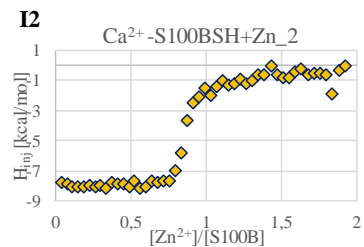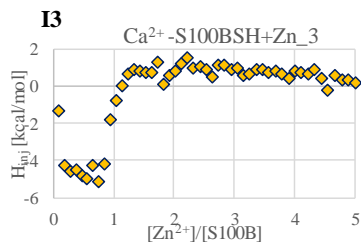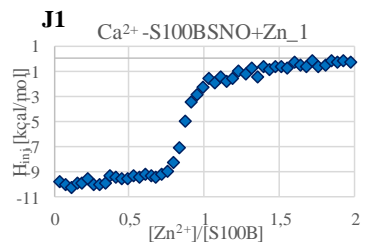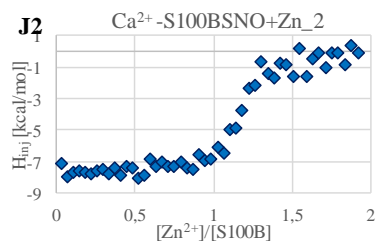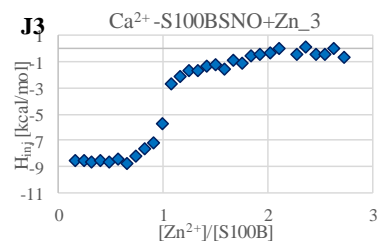

Supplement: S8 Fig — All ITC data (binding isothermograms) obtained for titration of: Ca2+ ions to S100BSH (A1-A7) and S100BSNO (B1-B3) protein solutions in TES buffer, pH 7.2, 15 mM NaCl at 25°C; Ca2+ ions to S100BSH (C1) and S100BSNO (D1) protein solutions in TES buffer, pH 7.2, 150 mM NaCl at 25°C; Zn2+ ions to S100BSH (E1-E3) and S100BSNO (F1-F3) protein solutions in TES buffer, pH 7.2, 15 mM NaCl at 25°C; Zn2+ ions to S100BSH (G1-G3) and S100BSNO (H1-H2) protein solutions in TES buffer, pH 7.2, 150 mM NaCl at 25°C; Zn2+ ions to Ca2+-S100BSH (I1-I3) and Ca2+-S100BSNO (J1-J3) protein solutions in TES buffer, pH 7.2, 15 mM NaCl at 25°C. (PDF) [file pone.0154822.s008.pdf]
