# Supplementary material for: An Interplay of S-Nitrosylation and Metal Ion Binding for Astrocytic S100B Protein
Source: PLoS One. 2016 May 9;11(5):e0154822. doi: 10.1371/journal.pone.0154822 (PMC4861259; doi:10.1371/journal.pone.0154822)
Supplement: S1 Text — (DOCX) [file pone.0154822.s009.docx]

**S1 Text. Materials and methods**

**Workflow of ITC experiments**

**S100BSH preparative protein purification**

(using RP-HPLC, detection at 220 nm and 280 nm, S2A Fig)

**Selective protein S-nitrosylation**

(by GSNO under native conditions, S2B Fig)

**Preparation of buffers and protein solutions for ITC experiments**

**Analytical RP-HPLC & mass spectrometry**

(to check purity of proteins, S3 and S4 Figs)

**Size exclusion chromatography**

(to assess oligomerization state, S6 Fig)

To achieve repeatability in each ITC experiment all protein solutions and buffers were precisely prepared according the following scheme (for each step a detailed description is presented):

**S100BSH preparative protein purification**

Protein purification was performed as described previously [1]. Briefly, the expression of S100B proteins in *E. coli* yields three forms of the protein for each of them due to variant N-terminal processing that yielded two additional forms, containing an N-terminal methionine or N-formylmethionine. Semi-preparative RP HPLC of protein fractions, lyophilized after phenyl-sepharose purification, allowed to separate the N-formylmethionine-containing proteins (S2A Fig, peak 2) from the other two forms (S2A Fig, peak 1) that were difficult to separate. The N-formylmethionine form was not used for any of the experiments presented in this paper. Protein concentrations were measured using HPLC as described below.

**Selective protein S-nitrosylation**

Protein S-nitrosylation was performed as described previously [1]. S-nitrosoglutathione (GSNO) was prepared by mixing 220 μl each of GSH in water and sodium nitrite in 0.1% TFA. The solution was incubated in the dark, under nitrogen for 10 min (final concentration of ∼100 mM GSNO) and used immediately after preparation. The final concentration of the GSNO stock solution was calculated from its absorbance at 334 nm. 0.5 mg of lyophilized, purified, reduced S100B (peak 1) was dissolved in 4 ml 50 mM Tris-HCl pH 8.0, with excess of CaCl_2_ (100 mM). After the addition of 52 μl of freshly prepared GSNO (100 mM), the reaction was kept for 10 min in the dark. The reaction mixture was then stopped and 1 ml of 0.25 M EDTA, pH 8.0 was added. The protein was purified using semi-preparative HPLC (80% yield after purification). Lyophilized protein was refolded and used for further experiments.

**Preparation of buffers and protein solutions for ITC experiments**

10 mM TES buffer, pH 7.2 with 15 mM or 150 mM NaCl was used for all ITC experiments. Lyophilized, purified S100BSH or S100BSNO proteins were initially dissolved in 10 mM TES buffer, pH 8.0, chelex-treated and dialyzed against 10 mM TES buffer, pH 7.2 for 1.5 h at 4°C immediately prior the ITC experiment. Protein concentration was determined using analytical HPLC (for details see below). The final protein concentrations span a range from 0.06 mM to 0.22 mM. CaCl_2_ titrant solutions were prepared in the same buffer as used for protein samples. ZnSO_4_ solutions were prepared in deionized water. Titrant concentrations used in all experiments span a range from 2.5 mM to 5 mM and from 0.5 mM to 1.25 mM, for CaCl_2_ and ZnSO_4_, respectively.

**Analytical RP-HPLC and mass spectrometry**

10 µL stock solution of protein was dissolved in 190 µL of 0.1% TFA and injected onto analytical HPLC column. The reversed-phase gradient for analysis was from 50 to 64% mobile phase (0.1% TFA in acetonitrile (v/v)) in 14 min (S3 Fig). The protein elution was detected by UV simultaneously at two different wavelengths either 220 and 280 nm for S100BSH or 220 nm and 334 nm for S100BSNO. The purity of protein was confirmed by mass spectrometry (S4 Fig). MS spectra were measured on a Q-TOF Premier mass spectrometer (Micromass). Desalted protein samples (HPLC peaks) were diluted 100 times in 0.1% formic acid, 50% acetonitrile/water solution or lyophilized and dissolved in the same solution before a syringe injection into the spectrometer source. For S-nitrosylated proteins and peptides, a typical cone voltage of 35 V at the mass spectrometer source was either reduced (20 V) or increased (80 V) to ensure the stability or to induce the dissociation of the S–NO bond, respectively. Raw spectra were deconvoluted to obtain the protein masses using Maxent 1 program (Micromass) [1].

**Size exclusion chromatography**

To determine oligomerization state od S100BSH and S100BSNO proteins size exclusion chromatography was performed. Briefly, 200 μL of 100 μM protein was injected onto Superdex 75 10/300 GL size exclusion chromatography column (GE Healthcare) in appropriate buffers used in ITC experiments (flow rate 0.5 ml/min). Analysis was performed before and after each ITC experiment (S6 Fig). The column was calibrated using commercially available mass standards as follows; albumin 67 kDa, ovoalbumin 43 kDa, chymotrypsinogen 25 kDa, ribonuclease 13,7 kDa (Amersham). 100 μg of each standard in 200 μl of ITC buffer was loaded onto column.

**Amino acid analysis of S100BSH or S100BSNO proteins**

Amino acid analysis was performed at BioCentrum Ltd. (Krakow, Poland). The protein samples were hydrolyzed in gas phase using 6 M HCl at 115°C for 24 h. The liberated amino acids were converted into phenylthiocarbamyl (PTC) derivatives and analyzed by high pressure liquid chromatography (HPLC) on a PicoTag 3.9 x 150 mm column (Waters, Milford, MA, USA).

**Determination of S100B protein concentration using HPLC**

The concentration of protein was determined using analytical reverse phase (RP)-HPLC by measuring peak area correlated with a standard protein sample previously determined via amino acid analysis. Namely, 10 µL of protein stock solution was dissolved in 190 µL of 0.1% TFA, injected onto analytical HPLC column and separated as described above. Protein concentration was calculated using A220 and the area of the calibration peak (200µL of 10 µM protein ≈ 1.116 107 area units).

**Determination of the residual calcium content**

A specific Ca^2+^ - chelator (1,2-bis(o-aminophenoxy)ethane-N,N,N',N'-tetraacetic acid) - BAPTA was used to determine residual calcium content in all protein solutions prior to ITC titrations. The measurement was performed using Cary-50 UV-Visible spectrophotometer (Varian). 20 μM BAPTA solution was prepared in the Ca^2+^ free buffer. The exact chelator concentration was determined by measuring of the Ca^2+^-dependent UV absorbance spectra of BAPTA at 297 nm (Ca^2+^-saturated). The chelator concentration (CQ) was calculated as CQ = A297/ε. The value of ε at λ297 for BAPTA is 5000. The Ca^2+^ residual content in 20 μM BAPTA solution was determined by recording the absorbance at 254 nm (A1). Next, an excess of EDTA was added to the chelator solution and absorbance was measured at 254 nm (A2). Finally, an excess of CaCl_2_ was added to the solution and absorbance at 279 nm was measured (A3). The calcium concentration (CaQ) in the BAPTA solution was estimated as CaQ = CQ*(A2 – A1)/(A2 - A3). Equimolar amounts of protein and chelator were mixed to determine the Ca^2+^ residual content in protein solution. The difference between the content of calcium ions in the mixed solution to chelator only was the amount of residual Ca^2+^ in the protein solution.

**Numerical methods**

a. Binding equilibrium – sequence of N sites

The numerical models used assume that the macromolecule *M*, serves *N* sites binding ions in the sequence according to the following formula [2]:

$k_{i}=\frac{M_{i}}{M_{i-1}*x}$ (Eq. 1a)

where *M_0_* and *x* are the concentrations of the free macromolecule and ligand, respectively. *M_i_* is the concentration of the macromolecule binding exactly *i* ligands, and *k_i_* is the corresponding constant of complex stability. When the concentrations of free macromolecule, *M_0_*, and ligand, *x*, are known, all the binding equilibria could be easily resolved according to the formula

$M_{i}=M_{0}*k'_{i}*x^{i}$ (Eq. 1b)

where

$k'_{i}=\prod_{j=1}^{i} k_{j}$ (Eq. 1c)

The total concentrations of the ligand, *X_t_*, and macromolecule, *M_t_* are the sums over all the possible binding states, according to the formula:

$M_{t}=M_{0}*\left(1+\sum_{i=1}^{N} k'_{i}*x^{i}\right)$ (Eq. 2a)

$X_{t}=x+M_{0}*\sum_{i=1}^{N} i*k'_{i}*x^{i}$ (Eq. 2b)

For a sequence of *N* binding sites, the system of *N* equations of the type (1b) extended by equations 2a and 2b defines the binding equilibrium of the system. The concentration of free ligand, *x*, and free macromolecule, *M_t_*, could be formally separated, leading to the general formulas:

$X_{t}=x+\frac{M_{t}*\sum_{i=1}^{N} i*k'_{i}x^{i}}{1+\sum_{i=1}^{N} k'_{i}x^{i}}$ (Eq. 3a)

$M_{0}=\frac{M_{t}}{1+\sum_{i=1}^{N} k'_{i}x^{i}}$ (Eq. 3b)

The equation 3a could be easily resolved against *x*, giving the polynomial function of the order *N*+1, and then the concentration of the free macromolecule, *M_0_*, is determined by equation 3b. Thus for *N*=1, 2, 3 the binding equilibrium could be resolved analytically, while for the more complication system the equation 3a is to be resolved numerically against *x*. Since the models of the sequence of two and three independent sites were applied in the reported analysis, the equation 3a was resolved analytically according to the common formulas for roots of quadratic [3], cubic [4] and quartic [5] equations.

b. Binding equilibrium – the sequence of N binding sites accompanied by the n equivalent non-interacting sites

In that case the analysis leads to the final equation in the form:

$X_{t}=x+\frac{M_{t}*\sum_{i=1}^{N} i*k'_{i}x^{i}}{1+\sum_{i=1}^{N} k'_{i}x^{i}}+\frac{M_{t}*n*k*x}{1+k*x}$ (Eq. 3c)

where *k* describes the binding constant of the single independent site. The equation 3c was resolved analytically for a system of two sequential and *n* independent binding sites.

c. ITC data analysis

The heat released in the series of n succeeding ligand injections of the volume *dV* to the sample cell of the volume *V_0_* filled with the macromolecule solution, after the correction for the lost volume, is described by the equation:

 (Eq. 4)

Equation (4) agrees with the standard correction, *ΔQ^exp^* estimated for the heat released in the *n-*th injection according to the formula:

$\Delta Q^{exp}(n) = Q(n) + \frac{dV}{V_{0}} \left[\frac{Q(n) + Q(n - 1)}{2}\right] - Q(n-1)$ (Eq. 5)

where *Q(n)* is the heat experimentally measured upon *n*-th injection, *ΔV* is the volume injected from the syringe, and *V_0_* is a volume of the sample cell.

The theoretical value of the heat, *Q^calc^(n)*, released in the *n* succeeding injections equals:

 (Eq. 6)

where *N* indexes the types of the binding sites, *V_0_* is the volume of the sample cell, *ΔH_i_* is the heat of binding of the *i*-th type site, *n_i_* is a number of that sites on macromolecule surface, and *X_i_(n)* is the concentration of *i*-th type complex after *n* injections. The set of *N* latter values, *X_k_(n)* are to be calculated with the aid of equation 3, according to the known ligand and macromolecule concentrations after *n* injections, and the assumed values of stability constants, *k_i_*. Finally, the set of N values of *k_i_*, *n_i_*, *ΔH_i_* should be optimized to obtained the best agreement between the experimental and theoretical data. Generally, two types of the optimization procedures can be used: fitting either *Q^calc^(n)* to the experimental *Q^exp^(n)* values, or *Q^calc^(n)-Q^calc^(n-1)* to *ΔQ^exp^(n),* both by the means of any least-squares method, using *χ2* values calculated according to the formulas 7a or 7b, respectively.

 (Eq. 7a)

 (Eq. 7b)

The two above methods are not equivalent due to the propagation of the experimental errors. The equation 7b, strictly corresponds to the method used in original ITC software. In equation 7a the eventual bias in the beginning of the titration influences all the succeeding data, while in equation 7b each data point participates only once in *χ2* value. Thus, the error treatment favors method 7b. Contrary, both the stability of the optimization algorithm and its sensibility towards the starting values strongly favor method 7a. As the compromise, a superposition of the two above methods according to the formula 7c was used in the initial steps of optimization procedure, where the scaling factor of 0.03 was arbitrary taken as the lowest value sufficiently improving the stability of the optimization procedure.

 (Eq. 7c)

The process of fitting model (equations 3,1,6) to the experimental data (equations 4,5) by the means *χ2* (equation 7c) was carried out with the use of implementation of Marquardt-Levenberg non-linear least-squares algorithm [6] from gnuplot [7].

**REFERENCES**

1. Zhukova L, Zhukov I, Bal W, Wysłouch-Cieszyńska A. Redox modifications of the C-terminal cysteine residue cause structural changes in S100A1 and S100B proteins. Biochim Biophys Acta. 2004;6;1742(1-3):191-201.
2. http://planetmath.org/encyclopedia/QuadraticFormula.html
3. N. Tartaglia’s derivation of the cubic formula, ttp://planetmath.org/encyclopedia/CubicFormula.html; First time published: G. Cardano, Ars magna, Nurenberg 1545
4. Ferrari-Cardano derivation of the quartic formula, http://planetmath.org/ferraricardanoderivationofthequarticformula
5. Poznański, J., Wszelaka-Rylik, M., and Zielenkiewicz, W. (2005) HEW lysozyme salting by high-concentration NaCl solutions followed by titration calorimetry. *Biophys Chem* **113**, 137-144
6. Marquardt, D.W. (1963) An algorithm for least-squares estimation of non-linear parameters. *J. Soc. Ind. Appl. Math*. **11**, 431-441.
7. Williams, T., Kelley, C., Gnuplot version 4.6, Copyright^(C)^, 2013.
